# ZmpTAC12 binds single‐stranded nucleic acids and is essential for accumulation of the plastid‐encoded polymerase complex in maize

**DOI:** 10.1111/nph.13248

**Published:** 2015-01-19

**Authors:** Jeannette Pfalz, Ute Holtzegel, Alice Barkan, Wolfram Weisheit, Maria Mittag, Thomas Pfannschmidt

**Affiliations:** ^1^ Department of Plant Physiology Institute of General Botany and Plant Physiology Friedrich‐Schiller‐University Jena D‐07743 Jena Germany; ^2^ Institute of Molecular Biology University of Oregon Eugene OR 97403 USA; ^3^ Department of General Botany Institute of General Botany and Plant Physiology Friedrich‐Schiller‐University Jena D‐07743 Jena Germany; ^4^ University Grenoble‐Alpes F‐38000 Grenoble France; ^5^ CNRS UMR5168 F‐38054 Grenoble France; ^6^ CEA iRTSV Laboratoire de Physiologie Cellulaire & Végétale F‐38054 Grenoble France; ^7^ INRA USC1359 F‐38054 Grenoble France

**Keywords:** chloroplast biogenesis, gene expression, plastid‐encoded plastid RNA polymerase (PEP)‐polymerase, ssDN/RNA‐binding, *Zea mays* (maize), ZmpTAC12

## Abstract

- The plastid‐encoded plastid RNA polymerase (PEP) represents the major transcription machinery in mature chloroplasts. Proteomic studies identified four plastome‐ and at least ten nuclear‐encoded proteins making up this multimeric enzyme. Depletion of single subunits is known to result in strongly diminished PEP activity causing severe defects in chloroplast biogenesis.
- Here, we characterized one PEP subunit in maize, ZmpTAC12, and investigated the molecular basis underlying PEP‐deficiency in *Zmptac12* mutants.
- We show that the *ZmpTAC12* gene encodes two different protein isoforms, both of which localize dually in chloroplasts and nuclei. Moreover, both variants assemble into the PEP‐complex. Analysis of PEP‐complex assembly in various maize mutants lacking different PEP‐complex components demonstrates that ZmpTAC12, ZmpTAC2, ZmpTAC10 and ZmMurE are each required to accumulate a fully assembled PEP‐complex. Antibodies to ZmpTAC12 coimmunoprecipitate a subset of plastid RNAs that are synthesized by PEP‐dependent transcription. Gel mobility shift analyses with recombinant ZmpTAC12 revealed binding capabilities with ssRNA and ssDNA, but not dsDNA.
- Collectively these data demonstrate that ZmpTAC12 is required for the proper build‐up of the PEP‐complex and that it interacts with single‐stranded nucleic acids.

## Abstract 2

See also the Commentary by Peter Kindgren and Åsa Strand

## Introduction

Plastids are of endosymbiotic origin and still resemble today's cyanobacteria in many aspects. One prominent characteristic among them is their genetic system which consists of the plastid genome (the ‘plastome’) and a machinery for its inheritance and expression (Lopez‐Juez & Pyke, 2005). The plastome of vascular plants is *c*. 120–150 kbp in size and encodes a highly conserved set of *c*. 120 genes that are required to build up the photosynthetic apparatus and the plastid gene expression machinery (Sugiura, 1992). Although the coding capacity of the plastome is limited in comparison to the nuclear genome, its proper expression is crucial to plant development and growth (Pogson & Albrecht, 2011).

Plastid gene expression combines features derived from the cyanobacterial ancestor with mechanisms that evolved after incorporation into the eukaryotic host (Barkan, 2011). Similar to their prokaryotic counterparts, most plastid genes are organized into operons with ‐35 (TTGaca) and ‐10 (TAtaaT) ‐like *cis‐* elements in the promoter regions (Zhelyazkova *et al*., 2012). The plastome is transcribed by two RNA polymerases (RNAPs), a nuclear‐encoded single‐subunit phage‐type (NEP; Liere *et al*., 2011) and a multimeric eubacterial‐type plastid‐encoded (PEP) polymerase. The PEP core enzyme is formed by the products of the four plastid genes *rpoA*,* rpoB*,* rpoC1* and *rpoC2* (Hu & Bogorad, 1990; Pfannschmidt *et al*., 2000; Suzuki *et al*., 2004; Steiner *et al*., 2011). One hallmark of the PEP transcription machinery, however, is the recruitment of additional nuclear proteins that do not resemble bacterial proteins. These novel subunits have been identified in a series of proteomic studies defining subunit composition of highly purified PEP‐complex assemblies (Pfannschmidt *et al*., 2000; Suzuki *et al*., 2004; Steiner *et al*., 2011) and the ‘transcriptionally active chromosome’ (TAC); the latter is a protein fraction that is composed of several multimeric protein complexes which are functionally related to DNA and RNA metabolism (Pfalz *et al*., 2006; Melonek *et al*., 2012). In Arabidopsis, TAC preparations also included products of the plastid *rpo* genes, as well as 18 proteins termed plastid transcriptionally active chromosome proteins pTACs 1–18 (Pfalz *et al*., 2006). The proteins pTAC2, ‐3, ‐6, ‐10, ‐12, ‐14, MurE, FLN1, FSD2, FSD3 and TrxZ of the pTAC proteins were found to be tightly associated with the PEP enzyme (Suzuki *et al*., 2004; Steiner *et al*., 2011). Mutations in Arabidopsis and maize genes encoding these PEP‐associated proteins (PAPs) exhibited a strong inhibition of chloroplast biogenesis mainly owing to repression of PEP‐dependent plastid genes (Pfalz *et al*., 2006; Garcia *et al*., 2008; Myouga *et al*., 2008; Arsova *et al*., 2010; Gao *et al*., 2011; Steiner *et al*., 2011; Jeon *et al*., 2012; Yagi *et al*., 2012; Williams‐Carrier *et al*., 2014). By contrast, the expression of NEP‐dependent genes was elevated. This molecular phenotype is similar to that of Δ‐*rpo* deletion mutants of tobacco (Allison *et al*., 1996; Hajdukiewicz *et al*., 1997; De Santis‐MacIossek *et al*., 1999; Krause *et al*., 2000; Legen *et al*., 2002). However, mutants lacking other pTAC proteins (e.g. FLN2, pTAC4, pTAC5, pTAC9, pTAC16, pTAC18) have different molecular phenotypes, not related to deficient transcriptional activities (Pfalz & Pfannschmidt, 2013). For example, no or small changes in gene expression and a modest decrease in the chlorophyll content have been reported. A model has been put forward which explains the phenotypic effects described for mutants lacking PAPs as a consequence of a defect in PEP‐complex formation (Pfalz & Pfannschmidt, 2013).

In an independent study, the protein pTAC12 has also been identified in a phytochrome B‐mislocalization screen in Arabidopsis (Chen *et al*., 2010). The isolated defective allele was called *hemera* and induces the same ivory phenotype as the *ptac12* T‐DNA insertion allele. The mutant is devoid of early phytochrome responses and the protein appears to be located to the nucleus, where it acts in early phytochrome signaling. In complementation experiments it was able to partially rescue the mutant *rad23* allele in yeast, suggesting that it is likely involved in nuclear protein degradation. A subsequent study demonstrated that pTAC12/HEMERA interacts with photo‐activated phytochromes by direct protein–protein interaction promoting pTAC12/HEMERA accumulation in the light (Galvão *et al*., 2012).

These findings suggest that pTAC12 belongs to a group of dually targeted proteins (Krause & Krupinska, 2009) that act in plastids and the nucleus. The protein pTAC1 is another representative of this group and is also known as Whirly1 (Why1). It has been reported to play important roles in diverse cellular processes including transcriptional regulation (Desveaux *et al*., 2002; 2004), plastid genome stability (Maréchal *et al*., 2009), RNA splicing (Prikryl *et al*., 2008; Melonek *et al*., 2010) and signal transduction pathways (Isemer *et al*., 2012).

Here we describe the analysis of maize *ptac12* mutant alleles (*Zmptac12*) and investigate ZmpTAC12 protein localization in detail. Functional analyses focusing on the plastid‐localized form aim to specify its contribution to expression of PEP‐targeted genes. We show that ZmpTAC12 exists in two isoforms, both dual localized to plastids and the nucleus, and that, within plastids, both reside in the PEP‐complex. We identify ZmpTAC12 in association with a subset of RNAs and demonstrate that ZmpTAC12 exhibits both single‐stranded (ss) RNA and ssDNA‐binding activity, but not double‐stranded (ds) DNA‐binding. Studies of protein complexes reveal that *ptac12*, and other maize *ptac* mutants lacking specific PAPs (e.g. *Zmptac2*,* Zmptac10* and *ZmmurE*), fail to accumulate the full PEP‐complex.

## Materials and Methods

### Nucleic acids

A cDNA clone (ZM_BFb0227G12) was obtained from the Arizona maize cDNA project (http://www.maizecdna.org/). The sequences of PCR primers and hybridization probes are listed in Supporting Information Table S1 http://www.ncbi.nlm.nih.gov/pmc/articles/PMC2718276/-S1.

### Plant material and growth conditions

The recovery and basic phenotypic features of the mutants used here were described in the following publications: *hcf7* (Barkan, 1993); *ppr10‐2* (Pfalz *et al*., 2009); *Zmptac12*;* Zmptac2‐3*,* Zmptac10‐1/‐2* and *ZmmurE‐1/‐3* (Williams‐Carrier *et al*., 2010, 2014); *Zmwhy1‐1/‐2* (Prikryl *et al*., 2008); and *w2* (*ZmDNA‐polA‐w2‐mum2/w2‐Burnham*) (Udy *et al*., 2012). The inbred line B73 was used for RIP‐chip assays, cell fractionation experiments and PEP‐complex purification. Seedlings were grown in soil at 26–28°C in cycles of 16 h : 8 h, light : dark, and harvested between 7 and 10 d after planting. Expression analyses of photosynthetic complex subunits and ZmpTAC12 accumulation along the leaf gradient involved fully expanded second leaf blades (just above the ligule). The lowermost part of elongating second leaf blades (first 4 cm from the leaf insertion of 7 d‐old seedlings) was used for studies of protein complex assembly.

### Antibody production

A PCR fragment covering the amino acids positions 80–394 of the full ZmpTAC12 sequence was introduced into pet28b(+)(Novagen, Madison, WI, USA). ZmpTAC12 was heterologously expressed from the construct pet28b/*Zmptac12* in *Escherichia coli* (*E. coli*) by isopropyl β‐d‐1‐thiogalactopyranoside (IPTG) induction for 4–5 h at 21°C. Recombinant protein was purified by nickel affinity chromatography and used for antibody production in rabbits (Biogenes, Berlin, Germany). The antigen was used for affinity purification of the antiserum on a HiTrap NHS‐activated column (GE Healthcare Life Sciences, Waukesha, WI, USA). Cloning, expression and purification of the recombinant RpoA protein (1–261 aa) was carried out by GenScript (Piscataway, NJ, USA). Anti‐RpoA antibodies were raised in rabbits (Biogenes).

### Subcellular fractionation and protein analyses

Total leaf protein extracts were prepared and analyzed according to Barkan (1998). Chloroplasts and stroma were prepared as described previously (Schmitz‐Linneweber *et al*., 2005). Thylakoid treatments were performed according to a protocol described by Prikryl *et al*. (2008), including the non‐ionic detergents Triton X‐100 (1%) and Chaps (1%), and sonication (6 × 10 s on ice at 20% output; Sonopuls HD 2200, Bandelin, Berlin, Germany) before centrifugation. Stromal proteins sedimented through sucrose gradients were analyzed as described for Why1 (Prikryl *et al*., 2008) except that aliquots of the stroma fraction were treated with either 30 units AluI or 50 μg ml^−1^ RNase A in samples supplemented with protease inhibitor cocktail (Roche Applied Science, Mannheim, Germany) for 60 min at 37°C before centrifugation.

Nuclei (prepared as by‐product of the purification of intact chloroplast) were further isolated as described by Luthe & Quatrano (1980). Solubilization of nuclear proteins was performed in two sequential steps: (1) pelleted nuclei were resuspended in lysis buffer (containing 80 mM (NH_4_)_2_SO_4_,) and sonicated (6 × 10 s on ice at 20% output), and (2) nuclei obtained in step (1) were resuspended in lysis buffer with (NH_4_)_2_SO_4_ adjusted to 0.8 M. Equal volumes of pellets and supernatants were analyzed by immunoblotting.

### PEP‐complex enrichment, 2D‐BN‐PAGE and PEP‐complex assembly analyses

The enrichment procedure for PEP‐associated proteins used a two‐step chromatographic method followed by two‐dimensional blue native polyacrylamide (2D‐BN‐PAGE). Methods were established based on the procedure of Schröter *et al*. (2010).

Fractions containing the PEP‐complex were identified by immunoblotting with the anti‐pTAC12 antibody and enzymatically by determining the *in‐vitro* transcriptional activity according to Steiner *et al*. (2011). For 2D gel electrophoresis of the QS/HS peak fraction, first dimension was performed as BN‐PAGE in a 4.5–14% acrylamide gradient gel followed by SDS‐PAGE in a 10% acrylamide gel as second dimension. BN‐PAGE was performed as in Dietzel *et al*. (2011).

For PEP‐complex assembly experiments, leaves were ground in liquid nitrogen and proteins extracted with Triton X‐100 lysis buffer (100 mM Tris‐HCl, pH 7.3, 10 mM MgCl_2_, 25% glycerol, 1% Triton X‐100, 5 mM β‐mercaptoethanol and 1× protease inhibitor cocktail). Recovered protein complexes (*c*. 50 μg) were separated by BN‐PAGE in the first dimension on a gradient gel of 4.5–14% acrylamide. The protein complexes in the gel were subjected to immunodetection according to Wittig *et al*., 2006.

Sucrose gradient analysis of total leaf proteins used the same extraction protocol as described for PEP‐complex assembly experiments including sonication treatments (3 × 10 s on ice at 20% output) and removal of insoluble plant material by centrifugation at 4°C at 20 000 ***g*** for 30 min. Obtained homogenates were then run through sucrose gradients (10–30%) at 4°C at 280 000 ***g*** for 15 h.

### RNA analyses

RNA analyses were carried out as in Pfalz *et al*. (2009). RT‐PCR used the Qiagen‐Kit according to the manufacture's instructions. Analysis of nucleic acids that coimmunoprecipitate with ZmpTAC12 were performed as in Schmitz‐Linneweber *et al*. (2005). Samples were treated with either 70 U DNase I (supplemented with 80 U RNasin) or RNase A/AluI (35 U)/EcoRI (35 U) before immunoprecipitation.

### Expression and purification of recombinant MBP‐pTAC12

A sequence encoding ZmpTAC12 (amino acids 72–555) appended to the C‐terminus of MBP was introduced into Rosetta 2 (DE3) cells (New England Biolabs, Ipswich, MA, USA) and the recombinant fusion protein was heterologously expressed by isopropyl‐β‐d‐1‐thiogalactopyranoside (IPTG) induction. Purification on amylose resin was performed according to the manufacturer's instructions.

### Nucleic acid binding assays

Electrophoretic mobility shift assays (EMSAs) were carried out according to established techniques. RNA substrates were generated by *in vitro* transcription from synthetic DNA templates with an annealed T7 promoter oligonucleotide at the 5′ end. For DNA probes, oligonucleotides were end‐labeled with T4 polynucleotide kinase. Before EMSAs, substrates were electrophoresed in an 8% denaturing polyacrylamide gel and purified by gel extraction. In competition assays, different amounts of nonradiolabelled ssDNA and ssRNA were included in the reaction.

### Accession numbers

Sequence data from this article can be found in the Arabidopsis Genome initiative or GenBank/EMBL databases under the following accession numbers: ZM_BFb0227G12 (*Zmptac12*), At2g34640 (*Atptac12*), NP_001044373 (*Osptac12*), XP_002298838 (*Ptptac12*), XP_001783761 (*Ppptac12*), XP_003569910 (*Bd‐ptac12*), DAA64282 (*Zmptac2*), AFW84282 (*Zmptac10*), AFW87925 (*ZmmurE*), AFW59605 (*ZmDNApolA*) and AFW75491 (*ZmWhy1*).

## Results

### Dual targeting of maize pTAC12 isoforms to nuclei and chloroplasts

**Figure 1:**
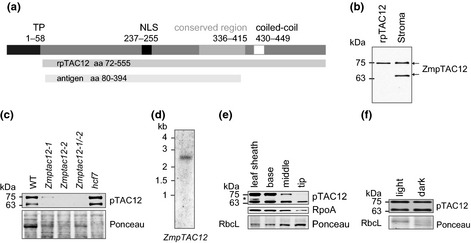
Schematic protein structure and expression of maize *ZmpTAC12* RNA and protein. (a) Predicted protein structure illustrating conserved region and domains. Recombinant proteins used for rabbit vaccination and production of rpTAC12 are indicated as light gray and dark bars, respectively. Numbers represent amino acids. TP, transit peptide, NLS, bipartite nuclear localization signal. (b) Immunoblot analysis demonstrating expression of two different isoforms of the ZmpTAC12 protein detected by affinity‐purified antibodies. Arrows denote both ZmpTAC12 proteins. Numbers on the left represent M_r_ of molecular weight standards. (c) Immunoblot analysis of ZmpTAC12. Ponceau staining is shown to demonstrate equal loading of protein samples (25 μg) taken from the second leaf. The *hcf7* mutant was used to control for pleiotropic defects. (d) *ZmpTAC12* transcript size determined by Northern blot analysis. Total leaf RNA (*c*. 50 μg) was hybridized to cDNA probes (Supporting Information Fig. S1). (e) Immunoblots (25 μg protein) showing ZmpTAC12 expression along the leaf developmental gradient of leaf 2 of a 10‐d‐old seedling. Shown image is representative of three independent experiments. The anti‐RpoA antibody served as control. Ponceau S staining is used to demonstrate equal loading of samples. The Ponceau staining of the membrane is shown below. Asterisk marks a band not detected in other immunoblots; its significance is not clear. (f) Immunoblots (25 μg protein) showing ZmpTAC12 expression in second leaves of light‐ and dark‐grown seedlings. The Ponceau staining of the membrane is shown below.

Genes homologous to the Arabidopsis *pTAC12* (*HEMERA*) gene have been found in all land plants, from bryophytes to angiosperms (Chen *et al*., 2010). Although the overall amino acid identity across phylogenetically distinct species is rather low (32–57% identity), a multi‐species alignment revealed a short, highly conserved region of unknown function (Fig. 1a, amino acids 336–415; Supporting Information Fig. S1). Most of the orthologs include a predicted coiled coil motif, which has been shown to mediate protein–protein interactions or homodimerization in other systems (Rose *et al*., 2004).

Two rabbit polyclonal ZmpTAC12 antibodies were generated using a 315 amino acid epitope as antigen (Fig. 1a). The affinity‐purified antibodies detected two ZmpTAC12 isoforms in the chloroplast stroma, with apparent molecular masses of *c*. 75 and 65 kDa (Fig. 1b). The identity of both was further confirmed by mass spectrometry (Table S2; Fig. S1). The larger variant corresponds in size to the predicted full‐length mature protein, as it co‐migrates with purified recombinant ZmpTAC12 (rpTAC12) protein lacking the predicted chloroplast transit peptide. Both forms are missing in *Zmptac12* mutants (Fig. 1c). To investigate potential variations in *ZmpTAC12* transcripts encoding the two isoforms, RNA gel blot analyses were performed. We detected only one RNA species, whose size matches that anticipated for the annotated full‐length *ZmpTAC12* transcript (Fig. 1d). The probes used were designed to hybridize with both potential *ZmpTAC12* variants if they were present (Fig. S2a). This result was further verified by RT‐PCR amplifying the complete coding region (Fig. S2b). In addition, a specific HA‐antibody recognized the two C‐terminally HA‐tagged ZmpTAC12 isoforms stably expressed in Arabidopsis by introducing the corresponding full length cDNA (Fig. S2c; see Supporting Information Methods S1 for details). Immunoblot analysis following treatments with protease inhibitors did not reveal varying accumulation pattern of the distinct isoforms (Fig. S2d). Thus, it seems reasonable to conclude that the smaller ZmpTAC12 form originates from posttranscriptional processes, such as alternative initiation of translation, differential proteolytic cleavage of the N‐terminal region, or post‐translational modification affecting protein mobility in the gel.

In order to determine whether the two isoforms accumulate differentially during leaf development, we analyzed the ZmpTAC12 accumulation profile along the natural leaf gradient of the developing maize leaf (Leech *et al*., 1973). Immunodetection of ZmpTAC12 in total leaf extracts demonstrated development‐dependent accumulation, with reduced concentrations at the leaf tip containing mature chloroplasts (Fig. 1e). This is in accordance with the accumulation profile of the RpoA subunit of the RNA polymerase, which was detected as control (Fig. 1e). Moreover, the ratio of the larger to smaller form decreases from the base (similar to leaf sheath) of the leaf blade to the tip. These changes in the accumulation patterns may be related to differential protein stability. Both isoforms accumulated to similar concentrations in light and darkness (Fig. 1f), unlike the Arabidopsis pTAC12 whose expression is light‐dependent (Chen *et al*., 2010; Galvão *et al*., 2012). These different expression patterns of pTAC12 (and other PAPs) might be related to the known differences of the photomorphogenic developmental programs in maize and *Arabidopsis* (Nemhauser & Chory, 2002). For example, seedling leaf development is light‐independent in maize but is light‐dependent in *Arabidopsis*.

**Figure 2:**
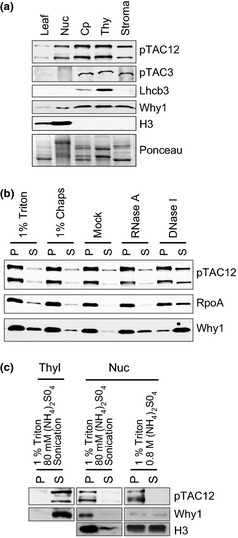
Dual localization of ZmpTAC12 to the plastids and the nucleus in maize. (a) Subcellular localization of ZmpTAC12. Protein extracts (25 μg) from leaves, nuclei (Nuc), chloroplasts (Cp) and plastid subfractions were resolved by SDS–polyacrylamide gel electrophoresis (PAGE). Proteins were detected by immunoblot analysis with the indicated antibodies. The Ponceau staining of the membrane is shown below. The photosystem II light‐harvesting protein Lhcb3, Whirly 1 (Why1) and the Histon 3 (H3) were used as marker proteins for thylakoids (thy), stroma and nuclei, respectively. Anti‐pTAC3 was used to monitor contaminations of nuclear fractions with TAC‐proteins. (b) Membrane association of ZmpTAC12. An equal proportion of pelleted thylakoids (as indicated above) and supernatant were subjected to SDS‐PAGE and examined by immunodetection with pTAC12 and RpoA antibodies. Anti‐Why1 was used as control for protein release. (c) ZmpTAC12 solubility. An equal proportion of pelleted material (as indicated above) and supernatant were subjected to SDS‐PAGE. Immunodetection was perform with pTAC12, Why1 and H3 antibodies. Proteins were isolated from 10‐d‐old maize seedlings. P, pellet; S, supernatant.

ZmpTAC12 is predicted to harbor a nuclear localization signal and an N‐terminal plastid‐directing transit peptide (Emanuelsson *et al*., 1999) (Fig. 1a). Indeed, the Arabidopsis protein was recently shown to be dually localized to chloroplasts and nuclei as observed by immunofluorescent labeling and immunoblotting (Chen *et al*., 2010). Furthermore, proteomic studies identified pTAC12 in chloroplast nucleoids in Arabidopsis and maize (Pfalz *et al*., 2006; Majeran *et al*., 2012). To address the localization of the two ZmpTAC12 isoforms experimentally we analyzed subcellular fractions from light‐grown seedlings by immunoblotting (Fig. 2a). Compared to the total leaf extract, both ZmpTAC12 isoforms were substantially enriched in chloroplasts. We also detected significant amounts in nuclei. Analysis of chloroplast subfractions localized ZmpTAC12 to both the stroma and chloroplast membranes. Purity of the chloroplasts and nuclei was tested by using antibodies raised against organelle‐specific proteins. To assess possible cross‐contamination of nuclei with plastid TAC‐proteins, fractions were analyzed with an antibody specific for pTAC3 (Yagi *et al*., 2012). The pTAC3 antibody detected a *c*. 100‐kDa protein only in chloroplasts, demonstrating that there is not significant contamination of the nuclear fraction with chloroplasts (Fig. 2a). These results provide evidence that ZmpTAC12 is dually localized to the nucleus and chloroplasts in maize, as in Arabidopsis. The nuclear located ZmpTAC12 isoforms appear to be the same size as the chloroplast proteins, where the longer variant has a size consistent with the full‐length mature form lacking the transit peptide. The fact that the nuclear form comigrates with the plastid form suggests that it may lack the N‐terminal transit peptide, implicating a mechanism for nucleus targeting different from that of classic NLS‐containing proteins.

In order to explore the basis for the association of ZmpTAC12 to the membrane, the membrane fraction was subjected to a variety of extraction procedures (Fig. 2b). Membrane‐association of ZmpTAC12 as well as of RpoA was resistant to low concentration of non‐ionic detergents (e.g. 1% Triton X‐100 and 1% CHAPS). DNAse I released a small portion (approximately one quarter) of ZmpTAC12 and RpoA to the soluble fraction. The majority of the control protein Why1, which is known to be associated with nucleoids and TAC, remained largely associated with membranes after washes with non‐ionic detergents but could be extracted from chloroplast membranes with DNase and RNase treatments, as shown previously (Prikryl *et al*., 2008). The lower sensitivity to nuclease treatments of ZmpTAC12, as compared to Why1, may be due to steric hindrance within plastid nucleoids, making the PEP‐complex less accessible to nucleases. Finally, plastidic ZmpTAC12 was released into soluble fractions after brief sonication of membrane pellets in buffer containing 1% Triton X‐100 and 80 mM (NH_4_)_2_SO_4_ (Fig. 2c). The same procedure, however, did not release large quantities of ZmpTAC12 from the nuclear pellet, which is consistent with the behavior of the nuclear proteins H3 and Why1. In the presence of high salt concentrations (e.g. 0.8 M (NH_4_)_2_SO_4_) *c*. 50% of H3 and Why1 were solubilized, but not ZmpTAC12. This is consistent with known differences in the solubility of nuclear proteins (Nováková *et al*., 2006). Together, these data support the identification of pTAC12 as a nuclear localized protein.

### Both ZmpTAC12 isoforms assemble into the PEP‐complex in maize

**Figure 3:**
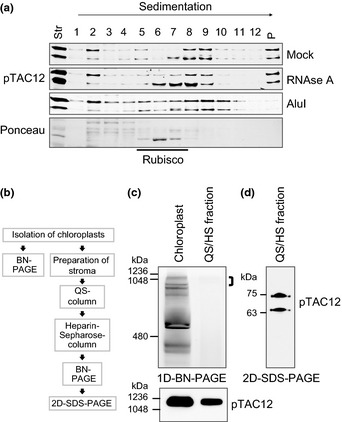
Both ZmpTAC12 isoforms are components of the PEP‐complex. (a) Sucrose‐gradient sedimentation demonstrating that ZmpTAC12 is associated in high molecular weight complexes containing nucleic acids. Stromal extracts of 7‐d‐old maize seedlings were sedimented through sucrose gradients after treatment with RNase A, or AluI, or incubated without nucleases (mock) using identical conditions. Equal proportions of gradient fractions were analyzed by immunoblotting with the indicated antibodies. Ponceau staining is shown to demonstrate the position of Rubisco (*c*. 550 kDa). Str, stroma; P, pelleted material. (b) Scheme of subcellular fractionation and purification. Intact chloroplasts were isolated from 10‐d‐old seedlings and lysed for stroma preparation. Transcriptionally active fractions were subsequently enriched by QS and HS chromatography, and analyzed by BN/SDS‐PAGE. (c) Blue native gel in the first dimension. Protein complexes from chloroplasts (50 μg crude chloroplast proteins, Cp) and the enriched QS/HS fraction were separated by BN‐PAGE (upper panel) and subsequently immunoblotted with the pTAC12 antibody (lower panel). Immunoblot corresponds to the upper part of the BN gel. Sizes of marker proteins are given on the left. (d) Immunoblot showing both ZmpTAC12 isoforms in PEP enriched fractions. The indicated gel strip (top bracket in (c); *c*. 1000 kDa) was incubated with denaturing buffer and a 10% SDS‐gel was run in the second dimension (2D‐SDS‐PAGE). ZmpTAC12 isoforms were examined by immunodetection with the anti‐pTAC12 antibody. Numbers on the left represent M_r_ of molecular weight standards.

In order to analyze the association of the two ZmpTAC12 isoforms with macromolecular complexes, stromal extracts were fractionated by sedimentation through sucrose gradients (Fig. 3a). Before centrifugation, stromal aliquots were treated either with RNase A or the restriction endonuclease AluI, or incubated under the same conditions but without enzyme treatments (Mock). Both ZmpTAC12 isoforms co‐sedimented over a wide range of fractions, with distinct peaks recovered in the size range of 800–1000 kDa and also in pelleted material at the bottom of the gradient (> 1200 kDa). AluI treatments eliminated the pelleted ZmpTAC12 material and increased the recovery of ZmpTAC12 in smaller particles, whereas RNase treatment had little effect. Notable, lower molecular weight fractions (fraction 3) contained predominantly the larger isoform that became more evident after AluI treatments. Nonetheless, the two isoforms generally behaved similarly during sucrose gradient sedimentation, suggesting that both pTAC12 isoforms are found in the same protein complexes.

In order to further clarify whether both maize variants reside in the PEP‐complex, ZmpTAC12 protein composition was investigated by a two‐step chromatographic method followed by 2D‐gel‐electrophoresis with a native first dimension, the established method for PEP‐complex purification (Fig. 3b). We detected both protein isoforms associated with the PEP‐complex purified from stromal extracts (Fig. 3c,d). Migration of ZmpTAC12 on BN gels showed that it resided in a complex of *c*. 1000 kDa (Fig. 3c), corresponding to the size reported for the purified full PEP‐complex in tobacco and mustard (Suzuki *et al*., 2004; Schröter *et al*., 2010).

### Genome‐wide analysis of nucleic acids associated with ZmpTAC12 *in vivo* suggests selective binding of subsets of PEP‐dependent genes

**Figure 4:**
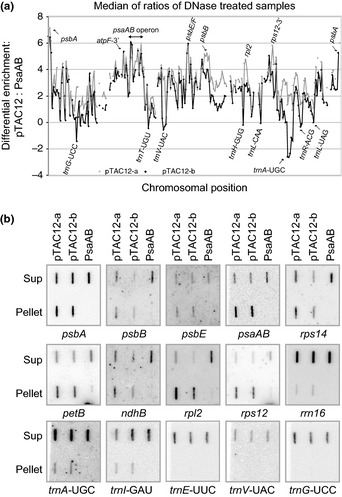
Coimmunoprecipitation assays identifying chloroplast RNAs (RIP) associated with ZmpTAC12 in maize seedlings. (a) Summary of RIP‐chip data. Stroma was treated with DNase I before immunoprecipitation. The median log2 (F635/F532) for replicate spots is plotted as a function of chromosomal position after subtracting the corresponding values for control immunoprecipitation with PsaAB antibody. The two ZmpTAC12 immunoprecipitations (a and b) used sera from different immunized rabbits. (b) Validation of RIP‐chip data by slot blot hybridization. Immunoprecipitations (with antibodies indicated above) and RNA extractions were performed as for RIP‐chip assays. One‐sixth of the RNA from each immunoprecipitation pellet and one‐twelfth of the RNA from the corresponding supernatant (Sup) were applied to replicate slot blots. Two replicate experiments were performed, using pTAC12 antibodies (a and b) from different immunized rabbits. RNAs purified from immunoprecipitations with antibodies against PsaAB were analyzed as controls. Blots were probed with the probes indicated below.

The observation that ZmpTAC12 co‐purifies with high molecular weight complexes containing DNA and possibly RNA prompted us to explore associated nucleic acid sequences using established RIP/DIP‐chip protocols for an initial screen (Prikryl *et al*., 2008). For RNA detection, the co‐immunoprecipitations were treated with DNase I to remove DNA from the samples (Fig. S2e,f). RNAs recovered in the pellet and supernatant were then labeled with different fluorescent dyes, and co‐hybridized to a tiling microarray of the maize chloroplast genome (Schmitz‐Linneweber *et al*., 2005). We calculated the median ratio of signal in the pellet vs supernatant for each spot on the microarray and compared those ratios to corresponding values from a control immunoprecipitation using antibody against PsaAB (a subunit of Photosystem I that is, not expected to bind RNA). Replicate experiments were performed with the two pTAC12 antibodies. A summary plot, presented in Fig. 4(a), reveals that the PEP‐dependent transcripts from the *psbA*,* psaA‐psaB‐rps14* and *psbE/psbF* loci are significantly enriched in the pTAC12 assays in comparison to the control. Most other RNAs known to be transcribed primarily by PEP were enriched among the sequences identified in the immunoprecipitate (e.g. *atpF/atpA*,* atpI/atpH*,* petL*,* psaC*,* psbJ/L/F/E*,* psbB/psbT/psbN* and others). Furthermore, high signal peaks were also detected for gene loci, which are targeted by both PEP and NEP‐polymerase (e.g. *ycf3*,* rps12*,* rpl20*,* rpl2, ndhB*,* ndhD*,* clpP* and others). By contrast, *rrn16* and some tRNAs (e.g. *trnG*‐UCC; *trnV*‐UAC; *trnA*‐UGC; *trnR*‐ACG, *trnL*‐UAA) are believed to be transcribed primarily by PEP (Kanamaru *et al*., 2001; Legen *et al*., 2002; Ishizaki *et al*., 2005; Williams‐Carrier *et al*., 2014), but these RNAs appeared not to be significantly enriched in the pTAC12 RIP‐chip experiments. The small enrichment peaks of latter genes could be a result of signal saturation caused by high RNA abundance in the supernatants. A summary of the pTAC12 RIP‐chip data is provided in Table S3.

The RIP‐chip data were validated by slot blot hybridization assays using the same coimmunoprecipitation conditions as for RIP‐chip assays (Fig. 4b). From the set of tested sequences, most of the highly enriched RNAs in the RIP‐chip assays (*psbA*,* psaAB*,* rps12*,* rpl2* and *petB*) were confirmed by the slot‐blot data; *psbB*,* rps14* and *psbE* coimmunoprecipitated with ZmpTAC12 to a relative moderate degree (*c*. 50%). Consistently with the RIP‐chip data, several tRNAs showed little or no enrichment. Taken together, these data provide support for the hypothesis that ZmpTAC12 associates with a subset of PEP‐derived transcripts, either directly or indirectly through its association with PEP.

In an attempt toward delineating plastid DNA loci associated with pTAC12 (and potentially loci of high PEP‐activity), DNAs were extracted from the immunoprecipitation pellet and supernatant and subjected to DIP‐chip analyses. In this assay, DNAs were fragmented including the restriction enzymes AluI and EcoRI before immunoprecipitation. Additionally, stromal extracts were treated with ribonucleases, and alkali hydrolysis was included to remove RNA after the immunoprecipitation. The signal ratio of the two DNA populations (pellet and supernatant) was calculated in order to reflect the enrichment of respective DNA segments precipitated by the pTAC12 antibodies (Fig. S3). Overall, nearly all of the input DNA was recovered in pelleted material. The most abundant sequences map to tRNAs, *matK* and genes encoding ribosomal subunits. Some of these were also identified among top ranked genes (e.g. *trnV*‐UAC, *matK*,* trnL*‐UAA and *trnG*‐UCC) using antibodies against the HA‐tagged RpoA subunit in tobacco (Finster *et al*., 2013). It is interesting, however, that DNA encoding tRNAs and ribosomal protein mRNAs appeared enriched, in comparison to their representation in the RIP‐chip data. These results suggest that ZmpTAC12, as an intrinsic subunit of the multimeric PEP‐complex, may associate preferentially with particular DNA regions, but additional experiments that include a crosslinking step would be required to clarify this point.

### ZmpTAC12 contributes to PEP‐complex formation

**Figure 5:**
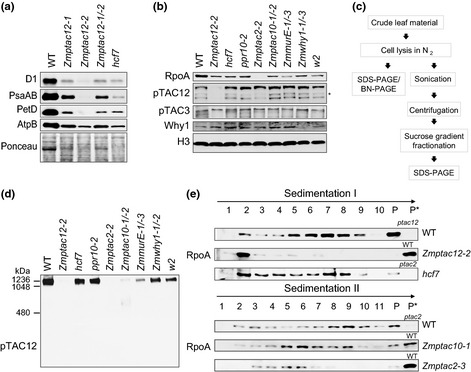
PEP‐complex assemblies in wild‐type (WT) and chlorophyll‐deficient maize mutants. (a) Immunoblot analyses of photosynthetic complex subunits. Labels on the left indicate the antibodies utilized for immunoblotting. Ponceau staining is shown to demonstrate equal loading of protein samples (25 μg) and abundance of RbcL (large subunit of Rubisco). (b) Accumulation of nucleoid‐associated proteins in chlorophyll‐deficient mutants (designation given above). Total proteins (25 μg) were separated by SDS‐PAGE and immunoblotted using antibodies against RpoA, pTAC12, pTAC3 and Why1. Histon H3 was used as loading control. Asterisk marks a band not detected in other immunoblots; its significance is not clear. (c) Flow chart of experimental procedure. (d) Separation of protein complexes from chlorophyll‐deficient mutants by BN‐PAGE and subsequent analyses of PEP‐complex assembly by immunoblotting with the anti‐pTAC12 antibody. Approximately 50 μg of total leaf protein (same preparations as above) from the base section of second leaves were loaded per lane. The gel was run in parallel with the SDS‐gel (shown in panel b) to ensure equal loading. (e) Sucrose‐gradient sedimentation of total leaf extracts from the basal half of the second leaf. Proteins from WT and indicated mutants were solubilized in Triton X‐100 protein lysis buffer with sonication, and soluble fractions were run through sucrose gradients. Fractions were collected and immunoblotted with an antibody against RpoA. Sedimentation I and II indicate two independent experiments. Proteins used in this experiments were isolated from 7‐ to 10‐d‐old maize seedlings. P, pelleted material; P*, pelleted material from WT or mutant leaf samples, respectively.

*Zmptac12* mutants were described previously to display a similar yellowish phenotype (Williams‐Carrier *et al*., 2010, 2014) as reported for the corresponding Arabidopsis mutants (Pfalz *et al*., 2006). Our allelic series of *Zmptac12* mutants includes mutants with various degrees of loss‐of‐function, reflected by a range of phenotypic severity. Here, we studied the molecular and biochemical defects associated with the *Zmptac12* allelic series (e.g. homozygous *Zmptac12‐1* and *Zmptac12‐2* mutants and the heteroallelic progeny *Zmptac12‐1/‐2*) in comparison with *hcf7* mutants, which have a moderate deficiency for plastid ribosomes (Barkan, 1993), and are similar in pigmentation and protein content to the *Zmptac12*. Defects in plastid gene expression parallel closely those previously shown for mutants with reduced PEP‐activity (Williams‐Carrier *et al*., 2014). Changes noted in *Zmptac12* include altered levels of PEP‐ and NEP‐dependent transcripts as well as low expression of photosynthetic enzyme complexes (Figs 5a,S4a). The most severe defects were observed in plants homozygous for the *Zmptac12‐2* allele, consistent with the position of this insertion within the protein‐coding region (Fig. S2a). Therefore, the latter was used in the following studies.

The nuclear localization of ZmpTAC12 suggests that it might function in phytochrome (PHY) signaling as reported for the *Arabidopsis* ortholog *ptac12/hemera* (Chen *et al*., 2010). To address its potential role in phytochrome responses during seedling development we examined the effect of light on mesocotyl elongation, which in maize is triggered by PHYB1 (and in part by PHYA and PHYC) in response to red light (Sawers *et al*., 2002; Sheehan *et al*., 2007). Similar light conditions repress coleoptile elongation in rice and hypocotyl elongation in *Arabidopsis* (Takano *et al*., 2005; Nagatani *et al*., 1993). Interestingly, *Zmptac12‐2* seedlings showed no apparent difference from the wild‐type (WT) in light‐dependent mesocotyl elongation (Fig. S4b). This experiment suggests that ZmpTAC12 is not required for phytochrome‐mediated signaling in developing seedlings. However, further experimental validation is required to put a more accurate figure on the nuclear role of pTAC12 in maize.

Plastid RNA synthesis is carried out mainly by the PEP‐transcription machinery, whose assembly and molecular regulation are still largely uncharacterized. To address the roles of specific subunits in the assembly of the complex, we examined the accumulation and assembly status of various PEP subunits in mutant lines lacking other subunits. For these experiments, we took advantage of maize mutants lacking PEP‐complex subunits pTAC12, pTAC2, pTAC10 or MurE (Williams‐Carrier *et al*., 2014) and a mutant deficient for Why1 (Prikryl *et al*., 2008). The latter was selected because it associates with plastid nucleoids but appears not to be an integral component of the PEP. In addition, the contribution of plastid DNA to the abundance and assembly of the complex was addressed by analyzing PEP assemblies in maize *w2* mutants, which have a *c*. 5‐fold decrease in cpDNA amounts due to a mutation in the gene encoding the plastid DNA polymerase (Udy *et al*., 2012). *Hcf7* with global defects in plastid translation, and *ppr10‐2* with specific defects in the expression of the ATP synthase and PSI (Barkan, 1993; Pfalz *et al*., 2009) served as controls to address effects of plastid gene expression defects on PEP‐complex assembly. First, protein abundance was investigated by immunoblot analysis. Because the buildup of the multimeric PEP‐complex occurs early during chloroplast development, total protein extracts were prepared from the base of second leaves. We observed a decline of the RpoA protein concentration in all mutants in relation to the amount in WT (Fig. 5b). RpoA was most severely reduced in *Zmptac2‐2* null mutants (Fig. 5b), but was only minimally decreased in the hypomorphic *Zmptac2‐3* allele (Fig. S5a). The total content of ZmpTAC12, ZmpTAC3 or WHY1 was unaltered in mutants lacking other PAPs (Fig. 5b). To determine the effect of each protein on assembly of the PEP complex, we explored by blue native PAGE analysis whether the assembly of the full complex was affected in mutants lacking specific PEP‐associated proteins. BN‐PAGE showed that *hcf7* and *ppr10‐2* mutants contained the full supramolecular PEP‐complex, and that this complex is slightly reduced in mutant alleles of *Why1* and *W2* (Fig. 5c,d). The impact of *W2* depletion is interesting, as it suggest that a reduction in cpDNA influences the amount of the complex, but the assembly is not impaired. By contrast, in mutants deficient in subunits of the PEP‐complex (*Zmptac2‐2* and *Zmptac10‐1/‐2*), pTAC12 failed to assemble into a complex of normal size (*c*. 1000 kDa; Schröter *et al*., 2010). This PEP deficiency was less pronounced in *ZmmurE‐1/‐3* mutants, which is most likely due to different strengths of the mutant alleles (Williams‐Carrier *et al*., 2014). Additional bands corresponding to the free, unassembled form or to smaller weight complexes were not detectable in any of the mutants, except for one very faint band in *Zmptac10‐1/‐*2. Perhaps the amount of distinct forms of ZmpTAC12 is below the detection limit in blue native gels. From these results, we conclude that pTAC2, pTAC10 and MurE are required for the accumulation of the full PEP‐complex and that an arrest in chloroplast biogenesis does not generally affect its stable assembly.

Next, we performed sucrose gradient sedimentation analysis to further evaluate the PEP‐assembly status in *Zmptac12‐*2 in comparison to that in the *hcf7* control and mutants lacking ZmpTAC2 and ZmpTAC10. For this experiment, crude protein extracts were sonicated in presence of low salt concentrations and Triton X‐100 to completely release thylakoid‐bound ZmpTAC12 to soluble fractions (note, the majority of the nuclear ZmpTAC12 is not released by this treatment). Solubilization efficiency and maintenance of assembled protein complexes were monitored by immunoblotting before sedimentation of the soluble supernatant through sucrose gradients (Fig. S5b–d). The status of PEP complex assembly was then assayed with antibodies against the PEP core (Fig. 5e). The bulk of RpoA protein in the *Zmptac12‐2* extract was found at the top of the gradients and was nearly undetectable in peak fractions compared to the WT. A strong defect in PEP complex formation was also observed in hypomorphic alleles of *Zmptac2* and *Zmptac10*. By contrast, parallel analysis revealed intact PEP assembly processes in the *hcf7* mutant, indicating that ZmpTAC12 as well as ZmpTAC2 and ZmpTAC10 are essential for the stable assembly of the core PEP polymerase. Interestingly, RpoA was detected in complexes of intermediate size in extracts from *Zmptac2‐3* and *Zmptac10‐1* mutants suggesting the buildup of partially formed complexes, whereas these complexes were not observed in the *Zmptac12‐2* mutant (Fig. 5e). ZmpTAC12 appears also to be part of lower molecular weight complexes that are most dominant in gradient fractions of similar size (Figs 5e,S5e). These studies show that ZmpTAC12, ZmpTAC2 and ZmpTAC10 are each required to assemble PEP into a high molecular weight complex, and suggest that the loss of PEP activity in these mutants is a consequence of these assembly defects.

### ZmpTAC12 interacts with ssRNA and ssDNA

**Figure 6:**
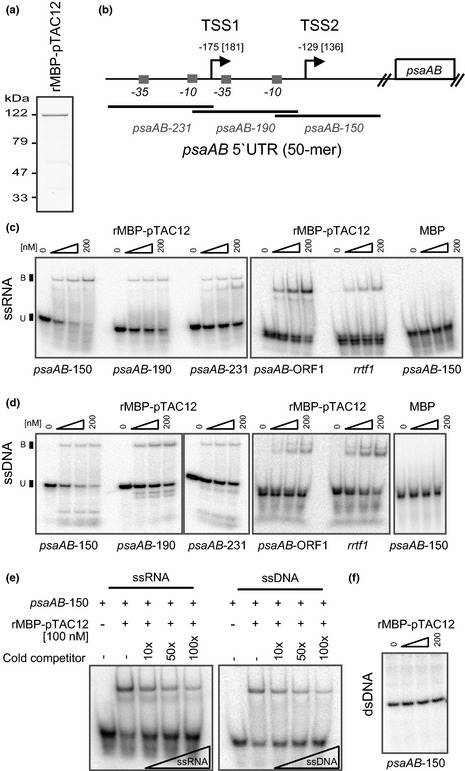
Nucleic acid binding activities of maize ZmpTAC12. (a) Purified recombinant rMBP‐pTAC12 after elution from amylose resin. Samples were analyzed by SDS‐PAGE and stained with Coomassie blue. (b) Schematic representation of *psaAB* locus. White box illustrates coding region. Position of ‐35 and ‐10 elements are depicted as small gray boxes. Bent arrows denote transcription start sites (TSS1 and TSS2) with the nucleotide positions relative to the ATG site of the rice gene and the corresponding position within the maize (in brackets) sequence, respectively. Transcription start sites (TSSs) and promoter elements were deduced by cross‐species sequence alignment of the *psaAB* promoter in rice (O *ryza sativa*; Chen *et al*., 1993), barley (*Hordeumvulgare*; Zhelyazkova *et al*., 2012), and *Arabidopsis* (Fey *et al*., 2005) with that of maize, of which the TSS has not been mapped (Supporting Information Fig. S6). The positions of probes used in assays are diagrammed below. (c) and (d) Gel mobility shift assays showing binding of rMBP‐pTAC12 to ssRNA and ssDNA, respectively. Binding specificity was examined with indicated radiolabeled 50mer RNA or DNA (*c*. 100 pM) and with increasing amounts of rMBP‐pTAC12 and MBP, respectively. MBP was used as control. (e) Binding activity in absence and presence (10–100‐fold molar excess) of the non‐radiolabelled competitor ssRNA or ssDNA (*psaAB*‐150). (f) Gel mobility shift assay of dsDNA. 100 pM of *psaAB*‐150 dsDNA was incubated with increasing amounts of rMBP‐pTAC12. The gel was run in parallel with ssDNA substrates shown in (d).

In the RNA coimmunoprecipitation experiments (Fig. 4), the *psbA* and *psaAB* RNAs appeared to be the most enriched RNAs. We next asked whether the recombinant ZmpTAC12 (rMBP‐pTAC12) exhibits intrinsic binding properties for sequences corresponding to the tri‐cistronic transcript *psaA*‐*psaB*‐*rps14* (Fig. 6a–f). For this purpose we used mobility‐shift experiments. Sequences being studied cover the 5′UTRs of *psaAB* beginning at each of two potential transcription start sites (TSS1 and TSS2), the upstream located prokaryotic‐like ‘‐35’ and ‘‐10’ promoter elements, and the coding region (Figs 6b,S6). A sequence within the 5′UTR of *rrtf1* (a nuclear gene encoding the *redox‐responsive transcription factor 1*) was used as control template. Recombinant ZmpTAC12 protein was expressed in *E. coli* as a maltose‐binding protein fusion (rMBP‐pTAC12) and incubated with single‐stranded (ss) RNA in the presence of high concentrations of heparin (1 μg μl^−1^) to reduce nonspecific binding. Binding activities to ssDNA was assessed in parallel. As shown in Fig. 6(c,d), ZmpTAC12 binds to both ssRNA and ssDNA. Overall, the binding abilities to particular nucleotide sequence were very similar, suggesting rather a nonspecific binding mode. However, comparing the amount of free nucleic acids in each reaction, a slightly higher preference appears for the site surrounding TSS2 within *psaAB* relative to the adjacent sites. The binding to this sequence was confirmed by competing with cold ssDNA and ssRNA of the same sequence (Fig. 6e). Thus, it might be possible that ZmpTAC12 possesses weak binding preferences for sequences or structures in the *psaAB‐150* substrate, but additional experiments that include several other substrates would be required to clarify this point. Furthermore, binding to the corresponding double‐stranded (ds) DNA sequence was tested. We did not observe any differences between the samples without and with increasing amounts of the recombinant protein (Fig. 6f), suggesting that ZmpTAC12 is only capable to interact with ss but not dsDNA.

## Discussion

The PEP core enzyme exhibits overall structural and functional similarities to the eubacterial transcription system (Liere *et al*., 2011). Over the past few years, however, biochemical and genetic studies have uncovered several nuclear encoded proteins that appear to have a fundamental role in PEP‐mediated transcription (Pfalz & Pfannschmidt, 2013). Although it is known that the genetic disruption of these subunits in Arabidopsis compromises PEP‐mediated transcription, the specific roles of these subunits in plastid transcription remain to be characterized. In this study, we have investigated the function of the dual targeted protein pTAC12 (PAP5) in maize, with emphasis placed on its role in chloroplasts.

### Role of ZmpTAC12 in plastid gene expression

The assembly of the PEP‐polymerase is a complex process involving at least 16 subunits encoded by both nuclear and plastid genes (Suzuki *et al*., 2004; Steiner *et al*., 2011). To date, very little is known about the order in which subunits assemble into the multimeric PEP‐complex. In mustard cotyledons, for example, developmental stage‐specific variants of the PEP complex with different subunit composition were identified (Pfannschmidt *et al*., 2000). This might delineate a possible order of PEP‐assembly during early light‐dependent chloroplast development, whereby the Rpo subunits generate the core complex (PEP‐B) and which then is coated by additional proteins building the larger PEP‐A complex (Steiner *et al*., 2011; Pfalz & Pfannschmidt, 2013). However, our analysis of PEP‐assembly in young leaf tissue detected PEP‐complex amounts in *hcf7* similar to that in WT but not or significantly reduced in mutants lacking PEP‐associated proteins (Fig. 5b–e). A proper assembly of the PEP was also observed in a mutant lacking the plastid ATP synthase (*ppr10‐2*) (Pfalz *et al*., 2009), in a mutant with reduced plastid DNA content (*w2*) (Udy *et al*., 2012), or in a mutant deficient for the nucleoid‐associated protein Why1 (Prikryl *et al*., 2008). Taken together, these data indicate that the absence of a multimeric PEP‐complex in *pap* mutants is not attributable to an arrest of chloroplast development. Furthermore, neither the *Zmptac12‐2* nor *hcf7* mutants accumulated PEP complexes of intermediate size, whereas the latter were present in the hypomorphic *Zmptac2‐3* and *Zmptac10‐1* mutants (Fig. 5e). This observation may reflect at which stage the PEP assembly process is impaired. However, the failure to accumulate the PEP core enzyme could also be due to the absence of the RpoC1 and RpoC2 subunits, despite high mRNA expression levels (Fig. S4a).

A regulatory function for PAPs has been widely suggested based on the PEP‐deficiency observed in the corresponding inactivation mutants (Pfalz *et al*., 2006; Garcia *et al*., 2008; Myouga *et al*., 2008; Arsova *et al*., 2010; Gao *et al*., 2011; Jeon *et al*., 2012; Yagi *et al*., 2012; Yu *et al*., 2012). However, deletions of most known regulators of chloroplast transcription (such as sigma factors) do not result in such strong phenotypes but, rather, compromise the expression of a few genes or have only minor impact on plastidial metabolism (Barkan, 2011; Lerbs‐Mache, 2011; Liere *et al*., 2011). The PEP‐deficiency in *pap* mutants, thus, is not a conclusive indication for PAPs being important regulators; however, it does not exclude it either. Interestingly, pTAC2, pTAC3, pTAC10 and pTAC14 possess predicted protein domains (PPR, MutS, SAP, S1 and SET) that typically function to mediate RNA or DNA metabolism, and thus potentially could control PEP activity by modulating the conformation of targeted DNA/RNA during transcription. Indeed, pTAC10 of *Nicotiana benthamiana* has been shown to convey transcription‐stimulating activities and to bind RNA *in vitro* (Jeon *et al*., 2012). Another factor important for transcriptional activity is pTAC3; it associates with PEP‐dependent transcribed regions in a light‐dependent manner and, thus, could be important for transcription initiation and elongation (Yagi *et al*., 2012), respectively.

### ZmpTAC12 associates *in vivo* with subsets of PEP‐derived transcripts

Our initial RIP‐chip analysis suggested that ZmpTAC12 associated with a subset of RNA sequences *in vivo*, whereas an association with specific plastid DNA sequences was only modestly evident. The latter observation is perhaps not surprising, considering the physical linkage to the PEP‐complex and nucleoids. Nevertheless, the enrichment peaks could represent positions of the PEP‐complex along plastid DNA, and thus, highlight regions of higher transcriptional activities. However, we found RNAs to be enriched in immunoprecipitates from throughout the chloroplast genome. The strongest enrichment signals map to genes containing either solely sequence motifs of bacterial promoters (e.g. *psaAB*,* psbA*,* psbE/F, psbB, atpF*) or along with NEP promoters (e.g. *rps12, rpl2*). These findings are supported by genetic analysis which uncovered characteristic features of PEP‐deficiency in *ptac12* mutants (Pfalz *et al*., 2006; Williams‐Carrier *et al*., 2014; Fig. S4). The high coverage of transcripts throughout the entire chloroplast genome is also consistent with the fact that the RNA majority originates from PEP activity. In green barley leaves, for example, at least 88% of all primary transcripts appear to be PEP‐transcribed (Zhelyazkova *et al*., 2012). Surprisingly, a few prominent PEP‐transcribed genes, such as *rrn16* and several tRNAs were only slightly or not enriched in immunoprecipitation pellets in RIP‐chip or slot blot assays. With respect to the overall enrichment, it seems possible that ZmpTAC12 associates with selected fractions of PEP‐derived transcripts, either directly or indirectly through its association with PEP. Furthermore, the results raise also the possibility that the PEP‐machinery might recruit proteins for specific RNA interactions to directly stabilize/process nascent transcripts or to couple mRNA to ribosomes, as emphasized by the finding of numerous RNA‐binding proteins co‐purifying with the nucleoid (Majeran *et al*., 2012). In this case, signals from immunoprecipitated DNAs and RNAs would produce different enrichment profiles. Reliable interpretation of RIP‐chip data for the highly abundant rRNAs and tRNAs, however, is difficult due to saturation of the probes and the fact that the bound form might represent only a minute fraction of the total. Therefore, the apparent absence of some tRNAs and rRNAs from the bound fraction should be interpreted with caution.

Our *in vitro* analysis of nucleic acid binding activities revealed that ZmpTAC12 interacts with both ssRNA and ssDNA but possesses only a weak binding preference for substrates with a particular nucleotide sequence or structure. These rather nonspecific binding properties are consistent with the large number of identified DNAs/RNAs in immunoprecipitates and might be linked to a general role in transcription (with multiple binding sites for PEP‐derived transcripts). As such pTAC12 could contribute to proper positioning of tethered DNA and/or RNA to active sites of transcriptional or post‐transcriptional processes, rather than being a specific modulator.

### Implication of dual‐targeting to chloroplasts and nuclei

Several features of the maize pTAC12 protein are similar to those reported previously for Arabidopsis in particular, its aforementioned subcellular distribution properties to nuclei and chloroplast. Interestingly, we immunodetected two ZmpTAC12 isoforms, each being dually targeted into both compartments (Fig. 2a). Proteins with multiple locations have been reported for a variety of phylogenetically distinct species (Carrie & Small, 2013; Krause & Krupinska, 2009). One frequent mechanism of dual localization involves alternative gene expression processes producing structurally different proteins from a single gene (Yogev & Pines, 2011). Other proteins possess sorting sequences for more than one compartment and consequently accumulate in forms of different molecular mass. If this holds true for ZmpTAC12, both the nuclear and plastid form should show clear differences in electrophoretic mobility in gels, considering the average size of a plastid transit peptide of *c*. 6 kDa. Controversially, our immunological analyses of subcellular fractions indicated that the nuclear ZmpTAC12 isoforms both have a similar molecular mass as the plastidic proteins (Fig. 2a), consistent with the localization data of pTAC12/Hemera in Arabidopsis (Chen *et al*., 2010). This finding is surprising as it points to a translocation mechanism toward the nucleus acting subsequently to the processing of the transit peptide within the plastids. Indeed, translocation from chloroplasts to the nucleus has been demonstrated for Why1 in transplastomic tobacco plants encoding the protein inside the chloroplasts (Isemer *et al*., 2012). For ZmpTAC12 the theoretical conjecture, however, still needs to be confirmed experimentally.

Furthermore, it remains to be investigated whether the detected ssRNA/ssDNA binding activities of ZmpTAC12 are active also in the nucleus. Although an action of the RAD23‐like multi‐ubiquitin binding activity appears unlikely in the plastids, because this degradation pathway does not exist in this compartment, an interaction of pTAC12 with nucleic acids within the nuclear compartment is highly likely. Early phytochrome signaling events seem not to be pTAC12‐dependent in maize, but ZmpTAC12 nevertheless could be involved in phytochrome signaling at a later stage of development.

## Supporting information

Please note: Wiley Blackwell are not responsible for the content or functionality of any supporting information supplied by the authors. Any queries (other than missing material) should be directed to the *New Phytologist* Central Office.

**Fig. S1** Genes homologous to *ZmpTAC12* are found in land plants.
**Fig.S2** *ZmpTAC12* gene encodes two different protein isoforms.
**Fig.S3** Coimmunoprecipitation assays identified chloroplast DNAs (DIP) associated with maize ZmpTAC12.
**Fig. S4** Transcript accumulation of plastid encoded genes and phenotypes of wild‐type and *Zmptac12‐2* seedlings grown under different light conditions.
**Fig. S5** Release of thylakoid‐associated maize ZmpTAC12 fraction by sonication and analyses of PEP‐complex assembly.
**Fig. S6** Comparison of the *psaAB* promoter sequences from maize (Zm), rice (Os), barley (Hv) and *Arabidopsis* (At).

**Table S1**List of primers used in this study
**Table S2** Liquid chromatography‐electrospray ionization‐tandem mass spectrometry (LC‐ESI‐MS/MS) confirmed the identity the ZmpTAC12 protein
**Table S3** Top‐ranking fragments in ZmpTAC12 RIP‐chip assays

**Methods S1** Arabidopsis transformation with full‐length maize *ZmpTAC12* cDNA.
