# Supplementary material for: ZmpTAC12 binds single‐stranded nucleic acids and is essential for accumulation of the plastid‐encoded polymerase complex in maize
Source: New Phytol. 2015 Jan 19;206(3):1024–37. doi: 10.1111/nph.13248 (PMC6680207; doi:10.1111/nph.13248)
Supplement: Supplementary file 1 — Fig. S1 Genes homologous to ZmpTAC12 are found in land plants. Fig.S2 ZmpTAC12 gene encodes two different protein isoforms. Fig.S3 Coimmunoprecipitation assays identified chloroplast DNAs (DIP) associated with maize ZmpTAC12. Fig. S4 Transcript accumulation of plastid encoded genes and phenotypes of wild‐type and Zmptac12‐2 seedlings grown under different light conditions. Fig. S5 Release of thylakoid‐associated maize ZmpTAC12 fraction by sonication and analyses of PEP‐complex assembly. Fig. S6 Comparison of the psaAB promoter sequences from maize (Zm), rice (Os), barley (Hv) and Arabidopsis (At). [file NPH-206-1024-s001.pdf]

***New Phytologist* Supporting Information**

Article title: ZmpTAC12 binds single-stranded nucleic acids and is essential for accumulation of the plastid-encoded polymerase complex in maize

Authors: Jeannette Pfalz, Ute Holtzegel, Alice Barkan , Wolfram Weisheit, Maria Mittag, and Thomas Pfannschmidt

Article acceptance date: 19 November 2014

The following Supporting Information is available for this article:

Supporting information Figs S1-S6

Supporting information Figs S1-S6

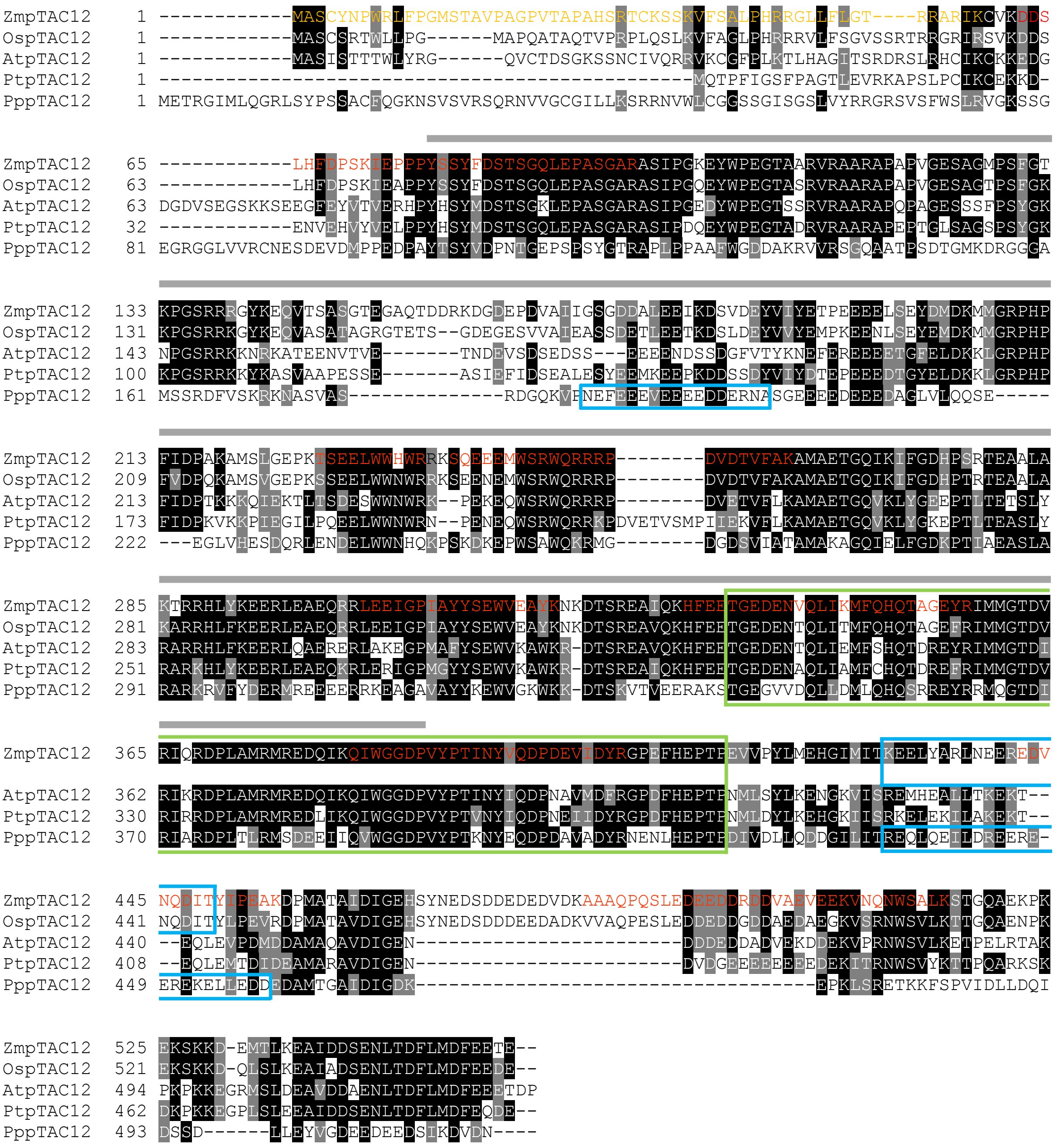

Fig. S1 Genes homologous to *ZmpTAC12* are found in land plants.

Multiple sequence alignment showing pTAC12 orthologs in maize (Zm), rice (Os), *Arabidopsis* (At), *Populus trichocarpa* (Pt), and *Physcomitrella patens* (Pp). Identical residues are shaded black and similar residues are shaded grey. Highly conserved region is boxed in green. The coiled coil domain was predicted using COILED-COILS (http://npsa-pbil.ibcp.fr/cgi-bin/npsa_automat.pl?page=npsa_lupas.html) and is boxed in blue. Orange sequence corresponds to the predicted plastid transit peptide. Identified peptides by mass spectrometry (Table S2) are highlighted in red. The grey line above the alignment marks the peptide used for antigen production.

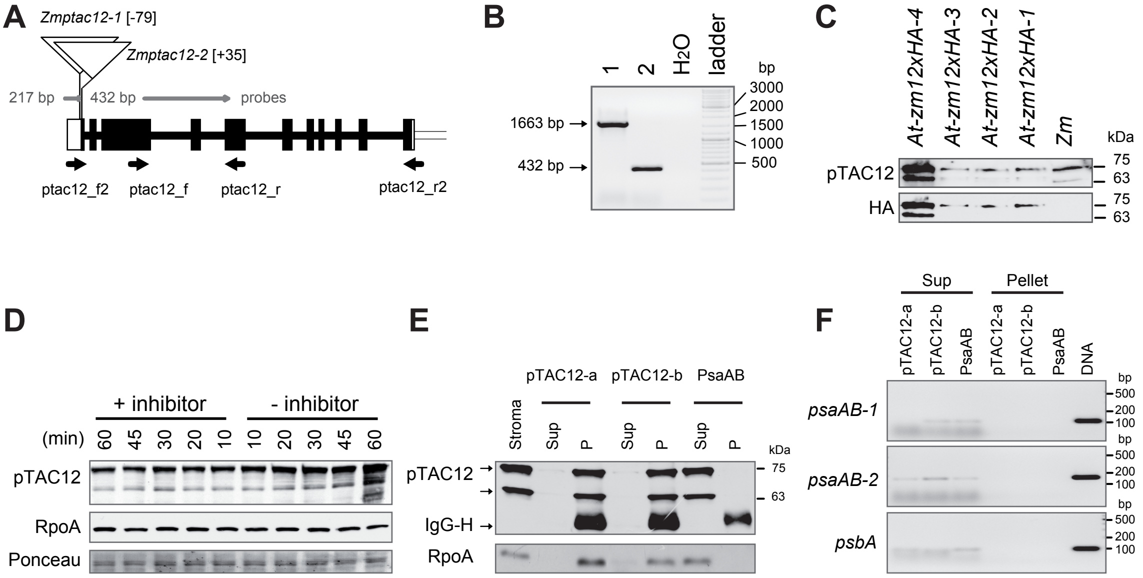

Fig. S2 Zm*ptac12* gene encodes two different protein isoforms.

A, Schematic representation of maize *ZmpTAC12* gene organization. Probes used for RNA blot hybridizations (grey arrowheads) and positions of RT-PCR primers (black arrows) are indicated on the genomic structure. B, RT-PCR to characterize *ZmpTAC12* transcripts. PCR was performed using cDNA synthesized from total RNA. *ZmpTAC12* full-length cDNA (1) and the middle segment (2) were generated using ptac12_f2/r2 and ptac12_f/r primers, respectively. DNA fragment was separated by 1% agarose gel electrophoresis followed by visualization with ethidium bromide. The sizes of calculated RT-PCR products are indicated with arrows and the numbers to the right of the gels indicate the size of the DNA ladder. C, Immunodetection of ZmpTAC12-HA stably introduced in *Arabidopsis* by agroinfection. The anti-pTAC12 antibody was used to detect ZmpTAC12 from total protein extracts (~25 µg) from *Zea mays* wild-type plants (*Zm*) and transgenic *Arabidopsis* (*At*) seedlings expressing ZmpTAC12-HA. An anti-HA antibody was used to confirm the expression of the HA-tagged ZmpTAC12 protein. *At-zm12xHA-1-4* are independent transgenic *Arabidopsis* lines expressing ZmpTAC12-HA at different levels. D, Immunodetection of ZmpTAC12 and RpoA in crude leaf extracts (25µg) from base sections of second leaves. Samples were assayed in presence or absence of protease inhibitors. E, Recovery of ZmpTAC12 in immunoprecipitations following DNase I treatment. Stroma proteins (~ 500 µg) were used for immunoprecipitation using protein A/G magnetic beads. Eluted proteins were resolved by 12% SDS-PAGE and subjected to immunoblot analysis with pTAC12 and RpoA antibodies. The antibodies used for immunoprecipitation are indicated above. The two bands specific to ZmpTAC12 are indicated by arrows. The prominent band at ~55 kDa corresponds to the IgG heavy (IgG-H) chain. Numbers to the right represent M_r of molecular weight standards. F, DNA depletion from coimmunoprecipitation pellets following DNase I treatment. Extracted nucleic acids from coimmunoprecipitates were tested by PCR for 35 cycles using *psaAB* and *psbA* primers. Genomic DNA (5 ng) was amplified in control reactions. Numbers to the right represent the base pairs (bp) of DNA fragments. Sup, supernatant; P, pellet; a and b, sera from two different immunized rabbits.

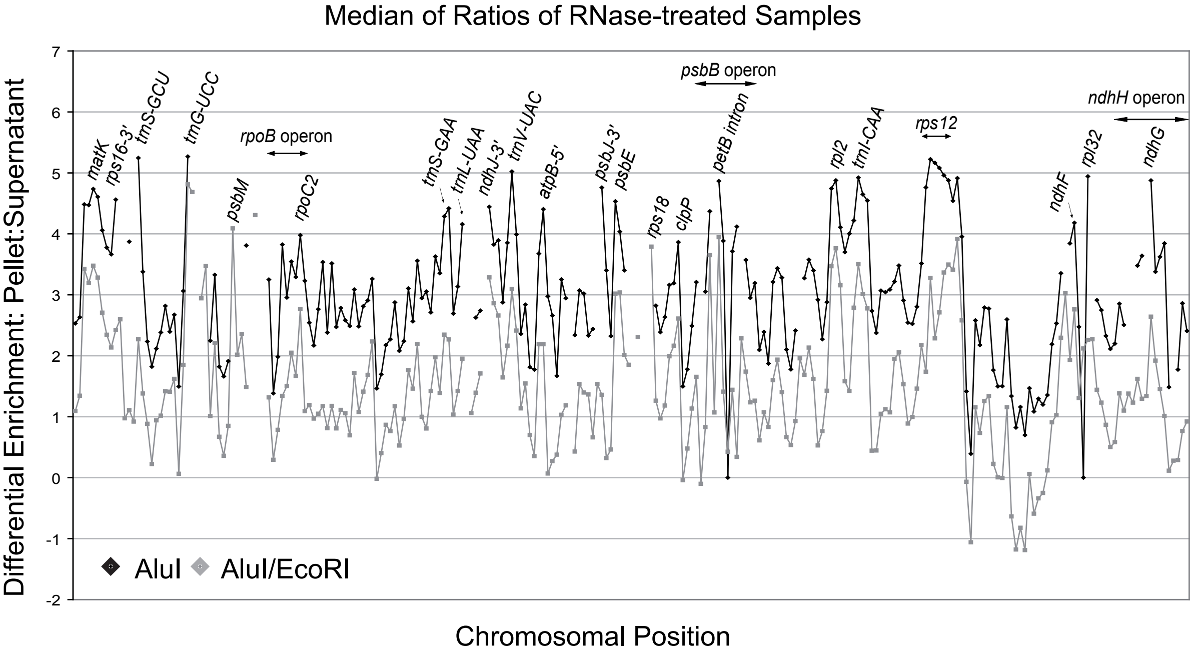

Fig. S3 Coimmunoprecipitation assays identified chloroplast DNAs (DIP) associated with maize ZmpTAC12.

Summary of DIP-Chip data. Stromal extract was treated with the restriction enzyme AluI/EcoRI (grey line) or AluI (black line) and RNAse A prior to immunoprecipitation. Nucleic acids recovered from immunoprecipitation were subjected to alkali hydrolysis to remove residual RNA prior to analysis by microarray hybridization. The ratio of signal in the pellet versus the supernatant (F635/F532) for replicate array fragments is plotted according to chromosomal position.

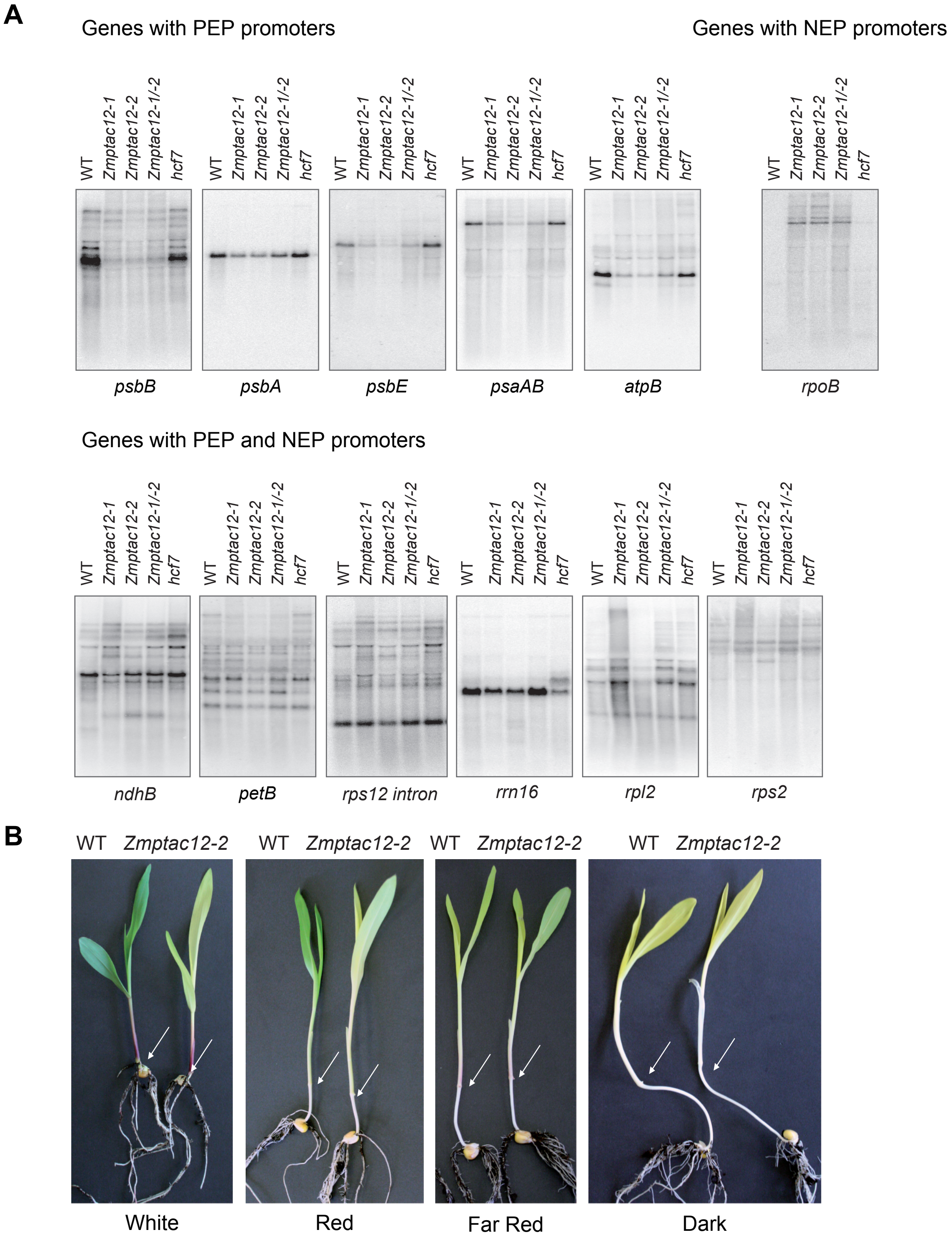

Fig. S4 Transcript accumulation of plastid encoded genes and phenotypes of wild-type and *Zmptac12-2* seedlings grown under different light conditions.

A, Isolated total RNA (10 µg) was subjected to RNA gel blot analyses and hybridized to DNA probes of indicated genes. B, Comparison of mesocotyl length in wild-type (left) and *Zmptac12-2* (right) grown under different light condition. Seedlings were grown in white (200 µmol m^{-2} s^{-1}) light or darkness, or under red (6 µmol m^{-2} s^{-1}) or far-red (2 µmol m^{-2} s^{-1}) for 7 days at 26-28°C. White arrows indicate boundary between mesocotyl and first leaf.

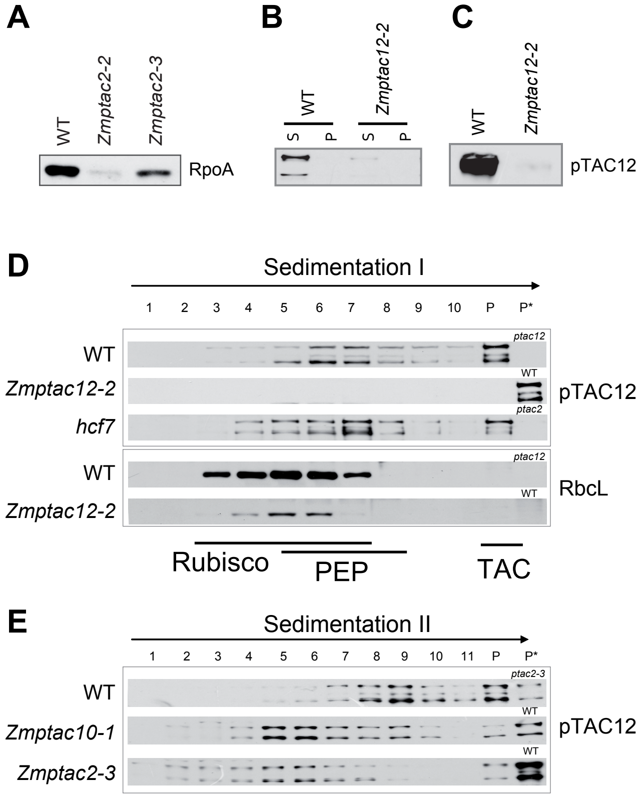

Fig. S5 Release of thylakoid-associated maize ZmpTAC12 fraction by sonication and analyses of PEP-complex assembly.

A, Immunoblot analysis of RpoA in wild-type and *Zmptac2* mutants. B, Immunoblot of a total protein fraction after sonication. Proteins were isolated from total leaf tissue of WT seedlings and sonicated to release ZmpTAC12 from thylakoids. Approximately 25 µg of protein were loaded per lane. P, pellet; S, supernatant. C, PEP-complex assembly detection by BN-PAGE and subsequent immunoblotting with anti-pTAC12. Approximately 50 µg of sonicated protein fractions from WT and *Zmptac12-2* seedlings were loaded per lane. D, E Sucrose-gradient sedimentation analysis. Total leaf proteins from basal half of second leaf were solubilized in Triton-X-100 protein lysis buffer with sonication and soluble fractions subjected to sucrose gradient centrifugation for analysis of PEP-complex assembly. Fractions were collected and immunoblotted with antibodies against pTAC12, and Rubisco (RbcL). Respective fractions containing Rubisco, PEP (plastid-encoded RNA polymerase), and TAC (transcriptionally active chromosome) are indicated by bars; P, pelleted material; P*, pelleted material from WT or mutant leaf samples, respectively.

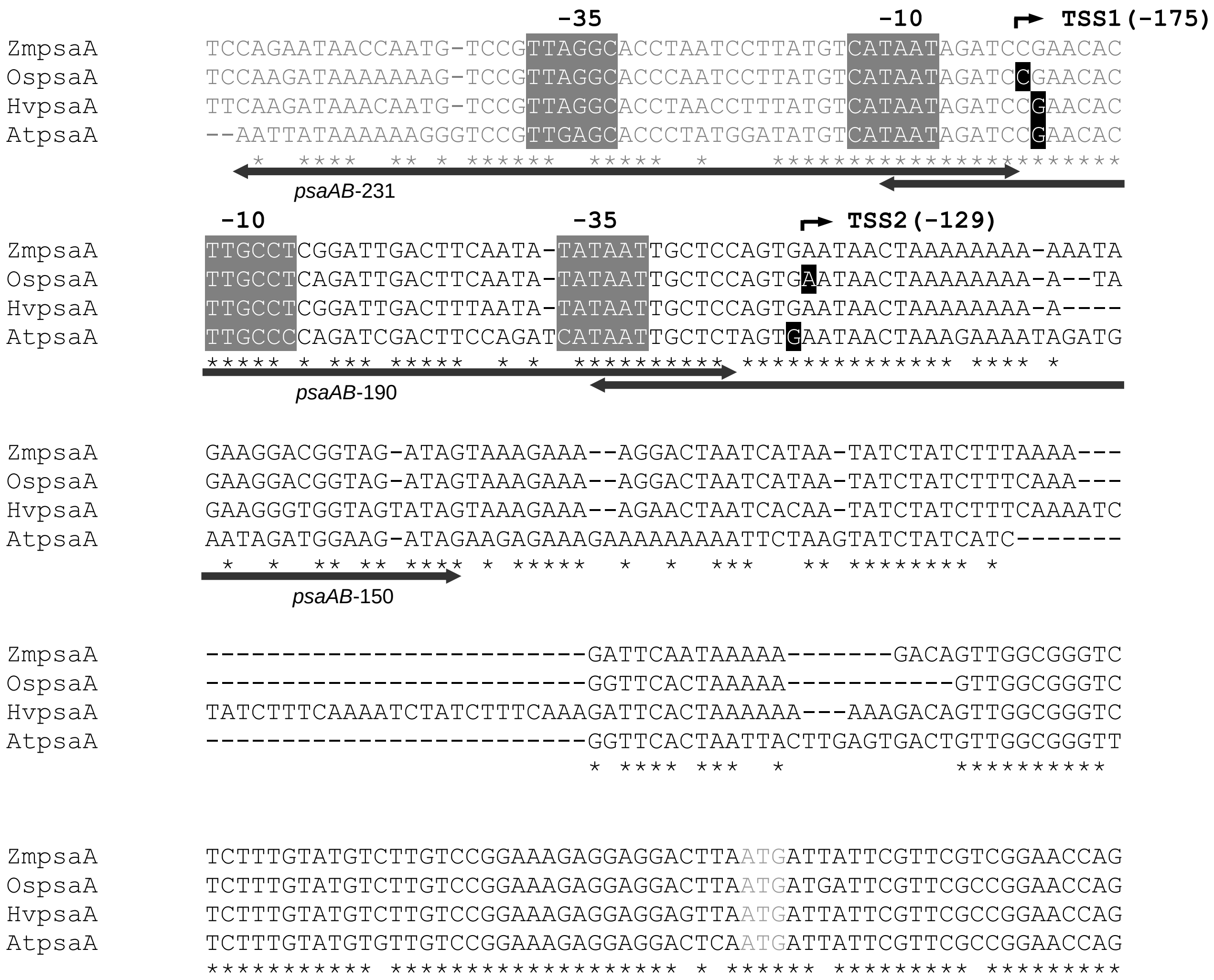

Fig. S6 Comparison of the *psaAB* promoter sequences from maize (Zm), rice (Os), barley (Hv) and *Arabidopsis* (At).

The known transcription start sites TSS1 and TSS2 (black shaded), translation start sites (ATG) and prokaryotic-like promoter elements (gray boxes) are indicated. Bent arrows denote mapped transcription start sites with the nucleotide positions relative to the ATG site of the rice gene (Chen et al., 1993). The positions of probes (double-headed arrows) used in binding assays are diagrammed below the sequence.

Chen MC, Cheng MC, Chen SC. 1993. Characterization of the promoter of rice plastid *psaA-psaB-rps14* operon and the DNA-specific binding proteins. *Plant Cell Physiol* 34: 577-584.
