# Supplementary material for: ZmpTAC12 binds single‐stranded nucleic acids and is essential for accumulation of the plastid‐encoded polymerase complex in maize
Source: New Phytol. 2015 Jan 19;206(3):1024–37. doi: 10.1111/nph.13248 (PMC6680207; doi:10.1111/nph.13248)
Supplement: Supplementary file 2 — Table S1List of primers used in this study Table S2 Liquid chromatography‐electrospray ionization‐tandem mass spectrometry (LC‐ESI‐MS/MS) confirmed the identity the ZmpTAC12 protein Table S3 Top‐ranking fragments in ZmpTAC12 RIP‐chip assays [file NPH-206-1024-s002.pdf]

Supporting Information Tables S1–S3

Table S1 List of primers used in this study.

Gene locus	Clone	Primer	Sequence
**pet28_ZmpTAC12**			
ZmpTAC12	ZM_BFb0227G12	ZmpTAC12_NdeI	5'-GCCACATATGCCCAGCTACTTCGAC-3'
		ZmpTAC12_SalI_k	5'-GGATCGTCGACCTAATTAATTGTTG-3'
**pMAL_ZmpTAC12**			
ZmpTAC12	ZM_BFb0227G12	ZmpTAC12_BglII	5'-GACCCGAGATCTATAGAACCGCCAC-3'
		ZmpTAC12_PstI	5'-TGTCCCTGCAGTTACTCGGTTTCC-3'
**pEnter221_Zm12-14**			
ZmpTAC12	ZM_BFb0227G12	Zmptac12-f-TOPO_1	5’-GGGGACAAGTTTGTACAAAAAAGCAGGCTCCATGGCGTCCTGCTACAACC-3’
		Zmptac12-r-TOPO_2	5‘-GGGGACCACTTTGTACAAGAAAGCTGGGTATGACTCGGTTTCCTCAAAGTC-3‘
**run on, slot blots and Northern**			
oligo probes			
psaAB		ZmpsaAB-150_r	5'-CTATCTACCGTCCTTCTATTTTTTTTTTTAGTTATTCACTGGAGCAATTA-3'
psaAB		ZmpsaAB-190_r	5'-GGAGCAATTATATATTGAAGTCAATCCGAGGCAAGTGTTCGGATCTATTA-3'
psaAB		ZmpsaAB-231_r	5'-GATCTATTATGACATAAGGATTAGGTGCCTAACGGACATTGGTTATTCTG-3'
psbA		ZmpsbA ORF1_r	5'-TGTTGCATATTCAGCTCCTGTTGCAGCTGC-3'
psbE		ZmpsbE ORF2_r	5'-GCATTACTATACCTTCCCTATTCATTGCGG-3'
trnA-UGC		ZmtrnA_UGC_r	5'-GGAGATAAGCGGACTCGAACCGCTGACATC-3'
trnG-UCC		ZmtrnG_UCC_r	5'-GAACCCGCATCGTTAGCTTGGAAGGCTAGG-3'
trnI-GAU		ZmtrnI_GAU_r	5'-GCCATCCTGGACTTGAACCAGAGACCTCGC-3'
trnV-UAC		ZmtrnV_UAC_r	5'-GAGTTGCTCTACCGAACTGAGCTATAGCCC-3'
trnE-UUC		ZmtrnE_UUC_r	5’-GAGAGATGTCCTGAACCACTAGACGATGGG-3’
PCR probes			
ptac12		Zmptac12_for2	5'-ACAACCTCCTTCCACGTGAG-3';
		Zmptac12_r3	5'-AATAGGCGCCACGGGTTGTA-3'
ptac12	ZM_BFb0227G12	Zmptac12_f	5'-CGTTCATTGATCCAGCAAAGG-3';
		Zmptac12_r	5'-ATTCCCCAGCAGTTTGGTGTT-3'
ptac12	ZM_BFb0227G12	Zmptac12_f2	5'-ATGGCGTCCTGCTACAACC-3';
		Zmptac12_r2	5’-CGGTTTCCTCAAAGTCCATAAG-3’
atpB		ZmatpBE_f	5'-TTTGATAGTCAAGAGTCGAGA-3';
		ZmatpBE_r	5'-TTTCCTTCACGAGTCCGTTC-3'
ndhB		ZmndhB_f	5'-CTTCAGCTTTAGCCACGCGAA-3';
		ZmndhB_r	5'-AATTGCAAGAATGGGGTTCA-3'
petB		ZmpetB_f	5'-GTCTTGAGATTCAGGCAATTGCAGATG-3';
		ZmpetB_r	5'-GCAGTAAGCAGAGGTAGTACAAAGGTATGT-3'
petG/psaJ		ZmpetG_f	5'-TCCCCTCGAACAAATCGAAATGATTGA-3';
		ZmpsaJ_r	5'-GGGAATGACAAAGCATCTGGGA-3'
psaAB		ZmpsaAB_f	5'-GGGTTTTCACAGTTTTGGCTT-3';
		ZmpsaAB_r	5'-CCCAAACATCCGACTGCATTT-3'
psbA		ZmpsbA_f	5'-CTTCTGCGGCGATAGGGTTGC-3';
		ZmpsbA_r	5'-CATTTTCAGTGGTTTCCCTGA-3'
psbB		ZmpsbB_f	5'-GCGGACGTTCCTTTTAGAAG-3';
		ZmpsbB_r	5'-GAGCGCCATGCCAAATGTGT-3'
psbE		ZmpsbE_f	5'-GTCTGGAAGCACGGGAGAACG-3';
		ZmpsbE_r	5'-TTCATCTAGTTGTTCTAAAGAA-3'
rpl2		Zmrpl2_f	5'-CCGTAGACAGGCAAGTGAAATC-3';
		Zmrpl2_r	5'-TATGTATGGCCGTGCCTAAG-3'

rpoB	ZmrpoB_f	5'-GGAATGGAAATGAGGGAATGTCCAC-3';
	ZmrpoB_r	5'-GACTCAATCCTCTTCTTCTCCTTAGCATTC-3'
rps2	Zmrps2_f	5'-GAAAGAGATGATAGAAGCGGGAGTTC-3';
	Zmrps2_r	5'-GAATTGAAGTCATAGTGTCATCGTTGGCAG-3'
rps12 exon	Zmrps12ex_f	5'-GCCCTTGTTGACGATTCTTTAC-3';
	Zmrps12ex_r	5'-GACTTTTCCACTATCAACCCC-3'
rps12 intron	Zmrps12in_f	5'-GGGTGGATCTCGAAAGATAGG-3';
	Zmrps12in_r	5'-GCCATGTGAATCGCTAGAAAC-3'
rps14	Zmrps14_f	5'-TCATTTGATTCGTCGATCCTC-3';
	Zmrps14_r	5'-GTATTTTATCCTTACCAACTG-3'
rrn16	Zmrrn16_f	5'-GGAGAGTTCGATCCTG-3';
	Zmrrn16_r	5'-GCTTTACGCCCAATCATTCC-3'
**RNA binding assays**		
T7 promoter	T7 promoter	5'-GAAACTAATACGACTCACTATAGG-3'
psaAB	ZmpsaAB-150	5'-CTATCTACCGTCCTTCTATTTTTTTTTTTAGTTATTCAC TGGAGCAATTACCTATAGTGAGTCGTATTAGTTTC-3’
psaAB	ZmpsaAB-190	5'-GGAGCAATTATATATTGAAGTCAATCCGAGGCAAGTG TTCGGATCTATTACCTATAGTGAGTCGTATTAGTTTC-3'
psaAB	ZmpsaAB-231	5'-GATCTATTATGACATAAGGATTAGGTGCCTAACGGAC ATTGGTTATTCTGCCTATAGTGAGTCGTATTAGTTTC-3'
**DNA binding assays**		
psaAB	ZmpsaAB-150	5'-TAATTGCTCCAGTGAATAACTAAAAAAAAAAAATAGAAG GACGGTAGATAG-3'
psaAB	ZmpsaAB-190	5'-TAATAGATCCGAACACTTGCCTCGGATTGACTTCAATA TATAATTGCTCC-3'
psaAB	ZmpsaAB-231	5'-CAGAATAACCAATGTCCGTTAGGCACCTAATCCTTATG TCATAATAGATC-3'

Table S2 Liquid chromatography-electrospray ionization-tandem mass spectrometry (LC-ESI-MS/MS) confirmed the identity the ZmpTAC12 protein.

Protein samples (PEP-complex preparation by a two-step chromatographic method) were separated by 10% SDS-PAGE and stained with Colloidal Blue (Staining Kit from Invitrogen GmbH). Bands were excised and the proteins *in gel* tryptic digested. Peptides were eluted and analyzed by LC-ESI-MS/MS according to Schmidt et al. 2006. For the data analysis, the Proteome Discoverer vs. 1.4 (Thermo Fisher Scientific Inc.) along with a database containing only the full length ZmpTAC amino acid sequence was used. The cut off XCorr criteria along with the false discovery rate of ≤ 1% were set according to Mann et al., 2014. The two N-terminal peptides that were only identified in the large ZmpTAC12 isoform (~75 kDa band) are highlighted in red.

[a] Peptide position in the ZmpTAC12 sequence

[b] Cross-correlation values

[c] Peptide charge

[d] Peptide identified in independent preparations (I and II)

Protein ID	Position[a]	Sequence	XCorr[b]	Charge[c]	Sample[d]
ZmpTAC12-S ~ 65 kDa	227-236	TSEELWWHWR	2,73	2	II
	251-261<br><br>252-261	RRPDVDTVFAK<br><br>RPDVDTVFAK	3,08<br>3,25<br>3,30<br>3,28	3<br>3<br>2<br>3	I<br>II<br>I<br>II
	303-320	LEEIGPIAYYSEWVEAYK	3,80	2	II
	332-346	HFEETGEDENVQLIK	5,50<br>5,68	2<br>2	I<br>II
	442-455	EDVNQDITYIPEAK	3,28<br>3,05	2<br>3	I<br>II
	507-515	VNQNWSALK	2,68<br>2,70	2<br>2	I<br>II
ZmpTAC12-L ~ 75 kDa	62-71	DDSLHFDPSK	3,08<br>2,67	2<br>2	I<br>II
	72-95	IEPPPYSSYFDSTSGQLEPASGAR	2,84	2	II
	227-236	TSEELWWHWR	3,04	2	II
	239-247	SQEEEMWSR	2,60	2	II
	251-261<br><br>252-261	RRPDVDTVFAK<br><br>RPDVDTVFAK	3,40<br>3,40<br>3,34	3<br>3<br>2	I<br>II<br>II
	303-320	LEEIGPIAYYSEWVEAYK	3,19	2	II
	332-346	HFEETGEDENVQLIK	5,82<br>5,60	2<br>2	I<br>II
	347-357	MFQHQTAGEYR	3,08	3	I
	382-406	QIWGGDPVYPTINYVQDPDEVIDYR	4,09	2	II
	442-455	EDVNQDITYIPEAK	3,05<br>3,69	2<br>2	I<br>II
	483-506	AAAQPQSLEDE EDDRDDVAEVEEK	3,09	3	I
	507-515	VNQNWSALK	2,86	2	II

Mann M, Hoppenz P, Jakob T, Weisheit W, Mittag M, Wilhelm C, Goss R. 2014. Unusual features of the high light acclimation of Chromera velia. *Photosynth Res* 122:159-69.

Schmidt M, Gessner G, Luff M, Heiland I, Wagner V, Kaminski M, Geimer S, Eitzinger N, Reissenweber T, Voytsekh O, Fiedler M, Mittag M, Kreimer G. 2006. Proteomic analysis of the eyespot of *Chlamydomonas reinhardtii* provides novel insights into its components and tactic movements. *Plant Cell* 18: 1908-1930.

Table S3 Top-ranking fragments in ZmpTAC12 RIP-chip assays.

[a] Fragments on the array are numbered according to chromosomal position.

[b] Median $\log_2$ (F635/F532) normalized across the experimental (anti-ptac12-a and anti-pTAC12-b) and control (anti-psaAB) immunoprecipitations. Replicate experiments constitute a total of *N* replicate spots above background.

anti-pTAC12-a IP				anti-pTAC12-b IP			
**Frag. #[a]**	**Fragment name**	**Median $\log_2$ ratio[b] (ZmpTAC12-PsaAB)**	**N[b]**	**Frag. #[a]**	**Fragment name**	**Median $\log_2$ ratio[b] (ZmpTAC12-PsaAB)**	**N[b]**
2	psbA2	7,14	6	1	psbA1	6,52	6
1	psbA1	7,14	6	2	psbA2	6,45	6
3	psbA3	6,16	6	71	rps14-1	6,08	6
249	IR/psbA	5,97	6	120	psbF/E	5,96	6
80	ycf3-3	5,81	6	72	rps14/psaB	5,89	6
192	rps12-3	5,80	5	77	psaA-2	5,44	5
120	psbF/E	5,69	6	65	atpF3	5,38	6
77	psaA-2	5,56	6	249	IR/psbA	5,27	6
119	psbJ/L/F/E	5,32	6	3	psbA3	5,26	6
136	psbB-1	5,22	6	119	psbJ/L/F/E	5,23	6
79	ycf3-2	5,18	6	80	ycf3-3	5,22	4
65	atpF3	5,17	4	189	ndhB/rps7	5,05	3
134	clpP/psbB	5,15	6	132	rps12int1-1	4,77	4
72	rps14/psaB	5,02	6	79	ycf3-2	4,49	4
138	psbB/psbT/psbN	4,98	5	195	rps125'	4,41	6
135	clpP	4,98	3	122	petL	4,39	6
195	rps125'	4,92	6	67	atpF/atpA	4,31	6
122	petL	4,91	5	13	psbK/psbI	4,17	6
232	ndhD-2	4,87	2	58	atpI/atpH	4,12	6
67	atpF/atpA	4,84	6	97	ndhK/ndhC	4,09	6
71	rps14-1	4,76	6	16	psbD1	4,07	6
169	rpl2int	4,75	5	131	rpl20	4,06	5
137	psbB-2	4,69	4	136	psbB-1	4,02	6
16	psbD1	4,63	6	137	psbB-2	3,99	6
75	psaB/psaA	4,60	5	75	psaB/psaA	3,97	5
76	psaA-1	4,60	5	235	psaC-2	3,97	6
235	psaC-2	4,58	6	81	ycf3-4	3,89	3
58	atpI/atpH	4,56	3	104	atpB-2	3,86	6
191	rps12-1	4,52	5	60	atpH2	3,86	6
13	psbK/psbI	4,44	6	133	rps12exon1	3,84	6
168	rpl2-1	4,44	6	192	rps12-3	3,83	4
194	rps12exon2	4,39	6	232	ndhD-2	3,77	3
193	rps12int2-1	4,37	5	112	ycf4-1	3,72	5
97	ndhK/ndhC	4,32	6	134	clpP/psbB	3,71	5
133	rps12exon1	4,24	6	36	psbM1	3,71	6
81	ycf3-4	4,22	5	63	atpFintron2	3,65	5

230	ycf5-2	4,21	3	191	rps12-1	3,62	4
189	ndhB/rps7	4,21	6	244	ndhAint	3,59	5
112	ycf4-1	4,20	4	78	ycf3-1	3,55	3
82	ycf3/trnS	4,16	4	68	atpA3'	3,53	5
8	rps16 intron	4,13	4	106	rbcL-1	3,52	6
132	rps12int1-1	4,12	4	61	atpF1	3,51	6
104	atpB-2	4,12	6	188	ndhB5'	3,50	2
131	rpl20	4,07	4	64	atpH/atpF	3,50	5
68	atpA3'	4,06	5	74	psaB-2	3,48	6
114	cemA	4,02	5	103	atpE/B	3,47	6
113	ycf4-2	4,02	2	82	ycf3/trnS	3,45	6
170	rpl2-2	4,01	4	66	atpA1	3,39	6
74	psaB-2	4,01	6	233	ndhD-3	3,38	4
106	rbcL-1	3,99	6	95	ndhJ/ndhK	3,37	5
103	atpE/B	3,97	4	135	clpP	3,37	6
59	atpH1	3,96	6	59	atpH1	3,35	6
245	ndhA-2	3,93	4	126	psaJ-2	3,34	6
60	atpH2	3,92	6	185	ndhB-2	3,31	3
17	psbD2	3,91	6	123	petE	3,28	6
57	atpI-2	3,86	4	96	ndhK	3,27	4
123	petE	3,85	6	18	psbC/trnS	3,25	6
40	petN3	3,84	5	116	petA-1	3,25	5
107	rbcL-2	3,83	6	230	ycf5-2	3,22	6
149	petDint-1	3,82	5	190	rps7-1	3,19	6
73	psaB-1	3,81	4	187	ndhBint-2	3,18	3
78	ycf3-1	3,81	6	56	atpI-1	3,15	6
188	ndhB5'	3,78	2	114	cemA	3,15	5
61	atpF1	3,77	5	156	rpl36/infA/rps8	3,13	4
244	ndhAint	3,75	2	57	atpI-2	3,10	5
127	psaJ/rpl33	3,73	2	138	psbB/psbT/psbN	3,10	6
213	rrn23-4	3,73	6	73	psaB-1	3,09	5
95	ndhJ/ndhK	3,71	4	186	ndhBintron	3,06	4
66	atpA1	3,71	4	17	psbD2	3,06	6
53	rpoC2/rps2	3,69	2	127	psaJ/rpl33	3,05	6
98	trnV-UAC-1	3,68	5	12	trnQ/psbK	3,01	6
121	psbE5'	3,60	6	121	psbE5'	3,00	6
214	rrn4.5	3,54	6	113	ycf4-2	3,00	3
12	trnQ/psbK	3,53	6	183	ndhB3'	2,98	4
156	rpl36/infA/rps8	3,52	4	170	rpl2-2	2,97	5
6	trnkintron	3,51	6	54	rps2-1	2,96	6
18	psbC/trnS	3,48	5	108	rbcL-3	2,92	6
64	atpH/atpF	3,47	6	107	rbcL-2	2,91	6
183	ndhB3'	3,47	6	168	rpl2-1	2,87	6
56	atpI-1	3,47	6	85	rps4	2,84	6
116	petA-1	3,46	3	245	ndhA-2	2,82	6
39	petN2	3,44	6	169	rpl2int	2,76	6
100	trnV-UAC-3	3,43	6	94	ndhJ-1	2,73	6
63	atpFintron2	3,41	4	166	rps19/orf75	2,71	6
140	psbH-1	3,40	6	160	rpl14	2,70	3
108	rbcL-3	3,39	6	5	matK	2,69	6
150	petD-3	3,37	6	164	rps3	2,69	2
234	psaC-1	3,36	5	213	rrn23-4	2,67	6
212	rrn23-3	3,32	5	128	rpl33	2,65	5

166	rps19/orf75	3,32	5	105	atpB5'	2,61	5
54	rps2-1	3,32	4	246	ndhA-3	2,57	2
221	ORF63//rps15	3,31	4	163	rpl16-int	2,57	3
94	ndhJ-1	3,30	5	172	rpl23	2,55	6
247	ndhA/ndhH	3,28	4	130	rps18/rpl20	2,53	2
163	rpl16-int	3,26	2	243	ndhA-1	2,50	2
118	psbJ3'	3,25	6	118	psbJ3'	2,50	4
203	trnI-GAU-1	3,24	6	248	ndhH	2,49	3
148	petB/D2	3,24	6	53	rpoC2/rps2	2,47	5
126	psaJ-2	3,24	6	8	rps16 intron	2,46	6
99	trnV-UAC-2	3,22	6	247	ndhA/ndhH	2,45	2
161	rpl16-1	3,19	5	109	psaI5'	2,41	6
171	rpl2/rpl23	3,15	5	234	psaC-1	2,40	6
220	ORF63	3,14	6	165	rps3/rpl22	2,39	2
69	trnR	3,14	6	157	infA	2,33	6
202	rrn16/trnI	3,14	6	171	rpl2/rpl23	2,33	5
102	atpE-1	3,12	6	6	trnkintron	2,30	5
139	psbB-psbH	3,12	3	102	atpE-1	2,30	6
144	petBintron	3,10	4	40	petN3	2,30	5
185	ndhB-2	3,06	2	194	rps12exon2	2,29	5
109	psaI5'	3,06	6	161	rpl16-1	2,27	6
190	rps7-1	3,04	5	176	orf241	2,27	2
164	rps3	3,02	3	212	rrn23-3	2,25	6
205	trnIint	3,01	6	9	rps16-1	2,24	5
125	psaJ-1	3,01	5	70	trnR/rps14	2,24	6
36	psbM1	3,00	6	220	ORF63	2,24	4
229	ycf5-1	2,98	6	214	rrn4.5	2,22	6
110	psaI-1	2,98	4	180	orf99/173	2,21	3
152	rpoA5'	2,98	4	219	trnN-GUU	2,16	6
141	psbH-2	2,98	4	125	psaJ-1	2,12	6
5	matK	2,96	5	229	ycf5-1	2,11	6
130	rps18/rpl20	2,95	5	140	psbH-1	2,09	6
44	rpoB1	2,94	4	7	rps16-2	2,09	6
181	orf173-3'	2,94	3	39	petN2	2,06	6
147	petD-1	2,94	5	173	rpl235'	2,04	5
157	infA	2,93	4	14	psbI	2,03	6
96	ndhK	2,93	6	231	ndhD-1	2,03	2
151	petD/rppoA	2,93	5	151	petD/rppoA	2,02	6
85	rps4	2,90	5	37	psbM2	2,00	6
204	trnI-GAU-2	2,85	6	141	psbH-2	2,00	6
159	rps8/14/16	2,83	5	69	trnR	1,99	6
172	rpl23	2,80	6	152	rpoA5'	1,98	5
37	psbM2	2,79	6	193	rps12int2-1	1,92	6
146	petB/D1	2,78	5	223	rps15/ndhF	1,83	6
211	rrn23-2	2,77	6	10	rps16-3	1,83	4
34	trnD-GUC5'	2,72	2	236	psaC/ndhE	1,75	6
26	trnG-UCC/ORF69	2,69	3	221	ORF63//rps15	1,74	5
145	petB-3	2,66	6	200	rrn16-1	1,73	6
25	trnG-UCC	2,64	6	21	ycf9/trnG	1,69	6
160	rpl14	2,64	4	142	petB-1	1,66	5
240	ndhG-2	2,63	5	110	psaI-1	1,64	5
70	trnR/rps14	2,59	6	27	ORF69	1,62	5
142	petB-1	2,52	4	199	rrn16-5'	1,58	6

173	rpl235'	2,51	4
124	trnW/trnP	2,49	6
128	rpl33	2,47	4
105	atpB5'	2,46	5
200	rrn16-1	2,44	6
215	rrn5-1	2,44	6
7	rps16-2	2,43	4
143	petB-2	2,36	5
197	orf58/orf85	2,33	4
20	ycf9	2,33	4
4	trnK1	2,29	6
236	psaC/ndhE	2,22	5
223	rps15/ndhF	2,18	6
180	orf99/173	2,18	3
10	rps16-3	2,17	6
158	rps8	2,17	2
209	trnA-UGC-2	2,13	6
115	cemA/petA	2,11	3
196	orf58-1	2,11	5
14	psbI	2,05	5
208	trnA-int	2,05	6
222	rps15	2,05	3
207	trnA-UGC-1	2,04	6
216	rrn5-2	2,00	6
199	rrn16-5'	1,96	6
49	rpoC1/C2	1,94	4
111	psaI-2	1,94	5
47	rpoB/rpoC	1,94	4
90	trnL-UAA-2	1,92	6
224	ndhF-1	1,91	3
219	trnN-GUU	1,90	6
30	trnT-GGU3'	1,86	4
24	trnfM-CAU	1,85	6
89	trnL-UAA-1	1,84	6
88	trnT-UGU2	1,79	6
227	rpl32	1,69	4
38	petN1	1,53	5
21	ycf9/trnG	1,52	6
218	trnR-ACG/orf23	1,49	6
41	trnC-GCA1	1,40	6
33	trnY-GUA	1,38	6
32	trnE-UCC	1,37	6
29	trnT-GGU	1,36	6
217	trnR-ACG	1,35	6
83	trnS-GGA	1,33	6
206	trnI/trnA	1,32	6
22	trnG-GCC	1,29	6
91	trnL/trnF	1,24	6
28	trnT-GGU5'	1,21	6
84	trnS-GGA2	1,18	6
42	trnC-GCA2	1,15	6
86	trnT-UGU	1,12	6
228	trnL-UAG	1,11	6

34	trnD-GUC5'	1,58	4
150	petD-3	1,57	6
222	rps15	1,57	5
139	psbB-psbH	1,56	6
225	ndhF-2	1,51	2
239	ndhG-1	1,50	2
237	ndhE	1,46	2
158	rps8	1,46	5
89	trnL-UAA-1	1,43	6
144	petBintron	1,42	6
211	rrn23-2	1,42	6
149	petDint-1	1,41	6
147	petD-1	1,37	5
181	orf173-3'	1,37	6
84	trnS-GGA2	1,34	6
143	petB-2	1,31	4
240	ndhG-2	1,30	5
44	rpoB1	1,30	6
215	rrn5-1	1,29	6
146	petB/D1	1,20	6
175	orf46/34/241	1,20	3
224	ndhF-1	1,16	5
148	petB/D2	1,13	6
111	psaI-2	1,13	4
115	cemA/petA	1,11	5
90	trnL-UAA-2	1,08	6
4	trnK1	1,08	6
47	rpoB/rpoC	1,07	3
124	trnW/trnP	1,07	6
49	rpoC1/C2	0,95	3
83	trnS-GGA	0,94	6
196	orf58-1	0,92	4
197	orf58/orf85	0,91	3
145	petB-3	0,90	6
226	ndhF/rpl32	0,88	2
203	trnI-GAU-1	0,83	6
88	trnT-UGU2	0,73	6
33	trnY-GUA	0,64	6
227	rpl32	0,62	6
29	trnT-GGU	0,59	6
38	petN1	0,57	5
30	trnT-GGU3'	0,56	6
22	trnG-GCC	0,56	6
216	rrn5-2	0,51	6
19	trnS-UAG	0,49	6
91	trnL/trnF	0,42	4
174	trnI-CAU	0,42	6
26	trnG-UCC/ORF69	0,40	6
202	rrn16/trnI	0,38	6
87	trnL-UAA1	0,36	6
24	trnfM-CAU	0,35	6
92	trnF-GAA	0,35	6
98	trnV-UAC-1	0,30	6

167	trnH-GUG	1,08	6
15	trnS-GCU	1,06	6
31	trnE/trnY	1,04	6
182	trnL-CAA	1,01	6
11	trnQ	0,97	6
87	trnL-UAA1	0,93	6
35	trnD-GUC	0,92	6
210	rrn23-1	0,89	6
23	trnG/trnfM	0,89	6
198	trnV-GAC	0,75	6
174	trnI-CAU	0,73	6
19	trnS-UAG	0,66	6
92	trnF-GAA	0,64	6
101	trnM-CAU	0,54	6

41	trnC-GCA1	0,27	6
23	trnG/trnfM	0,24	6
198	trnV-GAC	0,17	6
15	trnS-GCU	0,14	6
11	trnQ	0,12	6
205	trnIint	0,02	6
31	trnE/trnY	0,00	6
228	trnL-UAG	0,00	6
204	trnI-GAU-2	0,00	6
32	trnE-UCC	-0,03	6
42	trnC-GCA2	-0,04	6
182	trnL-CAA	-0,07	6
218	trnR-ACG/orf23	-0,10	6
101	trnM-CAU	-0,14	6
167	trnH-GUG	-0,21	6
28	trnT-GGU5'	-0,27	6
100	trnV-UAC-3	-0,28	6
35	trnD-GUC	-0,31	6
217	trnR-ACG	-0,33	6
86	trnT-UGU	-0,46	6
99	trnV-UAC-2	-0,56	5
209	trnA-UGC-2	-1,16	6
210	rrn23-1	-1,37	6
25	trnG-UCC	-1,45	6
208	trnA-int	-2,36	6
206	trnI/trnA	-2,62	6
207	trnA-UGC-1	-2,64	6
