# Supplementary material for: ZmpTAC12 binds single‐stranded nucleic acids and is essential for accumulation of the plastid‐encoded polymerase complex in maize
Source: New Phytol. 2015 Jan 19;206(3):1024–37. doi: 10.1111/nph.13248 (PMC6680207; doi:10.1111/nph.13248)
Supplement: Supplementary file 3 — Methods S1 Arabidopsis transformation with full‐length maize ZmpTAC12 cDNA. [file NPH-206-1024-s003.pdf]

Supporting Information

Methods S1 *Arabidopsis* transformation with full-length maize *ZmpTAC12* cDNA.

Vector construction

The ZmpTAC12-HA fusion construct (3xHA as C-terminal tag) used for *Arabidopsis* transformation was obtained by Gateway cloning into pGWB14 (Nakagawa et al., 2007). The coding sequences of *ZmpTAC12* was PCR amplified from maize cDNA (ZM_BFb0227G12), transferred into pDONR221 (pEnter221_Zm12-14), and recombined into pGWB14 (pGWB14_Zm12-3xHA). All plasmids *were sequenced* to *verify* that no errors *were* introduced.

Transgenic Plant Production

The pGWB14_Zm12-3xHA plasmid was introduced into *Agrobacterium tumefaciens* strain GV3101. *Arabidopsis* plants were transformed by the floral dip method (Clough and Bent, 1998). Transformants were selected on MS medium with sucrose supplemented with the 50 µg/ml hygromycin and allowed to self-pollinate. Seeds were surface sterilized, cold treated for 2 days, and germinated under controlled condition (21°C and a 16 h light / 8 h dark photoperiod). Total proteins for immunoblot analysis were isolated from leaves of 5-7 day-old seedlings.

Nakagawa T, Kurose T, Hino T, Tanaka K, Kawamukai M, Niwa Y, Toyooka K, Matsuoka K, Jinbo T, Kimura T. (2007). Development of series of Gateway binary vectors, pGWBs, for realizing efficient construction of fusion genes for plant transformation. *J Biosci Bioeng* 104: 34–41.

Clough SJ, Bent AF. (1998). Floral dip: a simplified method for Agrobacterium-mediated transformation of Arabidopsis thaliana. *Plant J* 16: 735-743.
